# Pharmacokinetics of rifampicin in adult TB patients and healthy volunteers: a systematic review and meta-analysis

**DOI:** 10.1093/jac/dky152

**Published:** 2018-04-26

**Authors:** K E Stott, H Pertinez, M G G Sturkenboom, M J Boeree, R Aarnoutse, G Ramachandran, A Requena-Méndez, C Peloquin, C F N Koegelenberg, J W C Alffenaar, R Ruslami, A Tostmann, S Swaminathan, H McIlleron, G Davies

**Affiliations:** 1 Department of Molecular and Clinical Pharmacology, Institute of Translational Medicine, University of Liverpool, Liverpool, UK; 2 Department of Clinical Pharmacy and Pharmacology, University Medical Center Groningen, University of Groningen, Groningen, The Netherlands; 3 Radboud University Medical Center, Nijmegen, The Netherlands; 4 Department of Biochemistry and Clinical Pharmacology, National Institute for Research in Tuberculosis, Chennai, India; 5 CRESIB, Barcelona Institute for Global Health, University of Barcelona, Barcelona, Spain; 6 College of Pharmacy and Emerging Pathogens Institute, University of Florida, Gainesville, FL, USA; 7 Department of Pulmonology, Stellenbosch University & Tygerberg Academic Hospital, Cape Town, South Africa; 8 Department of Pharmacology and Therapy, Universitas Padjadjaran, Bandung, Indonesia; 9 Department of Primary and Community Care, Radboud University Medical Centre, Nijmegen, The Netherlands; 10 Indian Council of Medical Research, New Delhi, India; 11 Division of Clinical Pharmacology, University of Cape Town, Cape Town, South Africa; 12 Institute of Global Health, University of Liverpool, Liverpool, UK

## Abstract

**Objectives:**

The objectives of this study were to explore inter-study heterogeneity in the pharmacokinetics (PK) of orally administered rifampicin, to derive summary estimates of rifampicin PK parameters at standard dosages and to compare these with summary estimates for higher dosages.

**Methods:**

A systematic search was performed for studies of rifampicin PK published in the English language up to May 2017. Data describing the *C*_max_ and AUC were extracted. Meta-analysis provided summary estimates for PK parameter estimates at standard rifampicin dosages. Heterogeneity was assessed by estimation of the *I*^2^ statistic and visual inspection of forest plots. Summary AUC estimates at standard and higher dosages were compared graphically and contextualized using preclinical pharmacodynamic (PD) data.

**Results:**

Substantial heterogeneity in PK parameters was evident and upheld in meta-regression. Treatment duration had a significant impact on the summary estimates for rifampicin PK parameters, with *C*_max_ 8.98 mg/L (SEM 2.19) after a single dose and 5.79 mg/L (SEM 2.14) at steady-state dosing, and AUC 72.56 mg·h/L (SEM 2.60) and 38.73 mg·h/L (SEM 4.33) after single and steady-state dosing, respectively. Rifampicin dosages of at least 25 mg/kg are required to achieve plasma PK/PD targets defined in preclinical studies.

**Conclusions:**

Vast inter-study heterogeneity exists in rifampicin PK parameter estimates. This is not explained by the available modifying variables. The recommended dosage of rifampicin should be increased to improve efficacy. This study provides an important point of reference for understanding rifampicin PK at standard dosages as efforts to explore higher dosing strategies continue in this field.

## Introduction

When it was introduced as part of combination therapy for TB in the 1960s, rifampicin revolutionized treatment and shortened the duration of therapy from 18 to 9 months. This would subsequently be shortened further to 6 months with the addition of pyrazinamide.^1^ Despite experience gained over the past five decades, the optimal dosage of rifampicin has not been established definitively. The current recommendation of 10 mg/kg in guidelines from the WHO has not changed since the introduction of rifampicin, at which time it was based on toxicological and financial concerns, with limited pharmacokinetic (PK) data available.^2^^,^^3^

For therapeutic drug monitoring (TDM) of rifampicin in TB treatment, a *C*_max_ of 8–24 mg/L (free plus bound drug) was suggested in the 1990s. This recommendation was based on a review of observed PK parameters and on expert opinion. Data from patients infected with HIV were not included.^4^^,^^5^ There was no pharmacodynamic (PD) component to the target, as MIC data were lacking in patient samples at that time. In the ensuing 20 year period, this original reference range was accepted as the target for rifampicin *C*_max_ in numerous studies addressing the utility of TDM for rifampicin.^6–11^ Treatment response is slow if rifampicin concentrations fall below this range.^12^^,^^13^

More sophisticated PK/PD analyses have since been performed on data from murine and human studies and there is a growing consensus that current dosages of rifampicin are inadequate; drug exposure appears scarcely to reach the upstroke of the dose–response curve.^14^ Accordingly, the target range of *C*_max_ for rifampicin TDM has been revised to emphasize the need to exceed 8 mg/L, rather than focus on an upper limit.^15^ At steady-state, drug exposure is thought to increase more than proportionally in response to modest dose increases.^16^ Increased dosages of rifampicin correlate with day 2 early bactericidal activity in a near-linear fashion in TB patients.^17^ There is an accumulating body of evidence demonstrating the safety and efficacy of higher-than-standard rifampicin doses in *in vitro*, animal and human studies and the adoption of this approach holds great appeal as a strategy to shorten TB treatment.^18–23^

Dose fractionation experiments have demonstrated that the PK/PD index most closely linked to rifampicin microbial kill is AUC/MIC, a finding corroborated by hollow-fibre models, which have additionally shown that *C*_max_/MIC is more closely linked to the suppression of resistance and the post-antibiotic effect.^20^^,^^21^ In TB patients, the 0–24 h AUC has a greater value than *C*_max_ or clinical features in predicting long-term clinical outcome.^24^

Scientific comparison of the findings of clinical trials investigating high rifampicin dosages requires an understanding of the PK parameters achieved with currently used dosages, so that the impact of dose escalation can be appreciated. For this reason, we conducted a systematic review and meta-analysis of published data describing rifampicin PK. As *C*_max_/MIC and AUC/MIC are the PK/PD indices best characterized, we focused on these PK parameters. The objectives of this study were: (i) to explore the inter-study heterogeneity in rifampicin PK; (ii) to derive summary estimates of rifampicin PK parameters at standard dosages; (iii) to compare these with summary estimates for higher-than-standard rifampicin dosages; and (iv) to contextualize these PK estimates using the available PD data.

## Methods

### Search strategy and selection criteria

Studies were identified in accordance with the Preferred Reporting Items for Systematic Reviews and Meta-Analyses (PRISMA) guidelines.^25^ PubMed, Scopus and MEDLINE electronic databases were searched. In PubMed and Scopus, titles and abstracts were searched using the terms ‘rifampicin’ OR ‘rifampin’ OR ‘antituberculous’ OR ‘antimycobacterial’ AND ‘pharmacokinetics’, to identify studies reported in the English language up to May 2017. The MEDLINE database was searched using the keywords ‘pharmacokinetic*’ OR ‘bioequivalence’ AND title words ‘rifampicin’ OR tubercul*’. Two reviewers (K. E. S. and G. D.) screened titles and abstracts for relevance and appraised full texts for inclusion in the meta-analysis using pre-specified selection criteria. Key articles were identified by consensus between K. E. S. and G. D. Prospective clinical studies were included if they collected PK data from adult patients with *Mycobacterium tuberculosis* infection and/or healthy adult volunteers receiving orally administered rifampicin.

Patients who received rifampicin for indications other than TB were excluded, because physiological fluctuations associated with different disease states are known to interfere with PK.^26^ Studies that collected data relating to paediatric populations were excluded, as were non-human studies, abstracts, reviews and correspondence. Papers reporting PK parameters derived from modelling analyses were excluded for several reasons: variability in modelling methods has the potential to introduce additional heterogeneity; over-parameterization of models can lead to statistical shrinkage and loss of data variability; and datasets are often reported in both modelling and non-compartmental analyses (NCAs), which would risk reporting some data in duplicate. Finally, studies assessing the impact of rifampicin on the PK of another drug, rather than reporting the PK of rifampicin itself, were excluded.

### Assessment of quality of studies

No validated tool exists to assess methodological rigour in PK studies. The priority is that samples are collected from subjects representative of target populations receiving dosage regimens of interest and relevance, rather than subjects who are randomized to one or other intervention. We considered this in our selection of studies, as well as ensuring that authors clearly described the pharmaceutical product, bioanalytical methods and statistical tools used.

### Data extraction

A data extraction form was designed and one reviewer (K. E. S.) extracted data from the included studies on the following items in addition to rifampicin PK parameters: study design; study population; sex; age; body weight; HIV status; treatment regimen; duration of treatment; rifampicin dose; whether rifampicin was administered as a separate drug or in a fixed-dose combination; whether dosing was daily or intermittent; PK sampling times; assay method; and data analysis method. These variables were selected *a priori* as it was felt that they were the factors most likely to impact rifampicin PK. Rifampicin was considered to be at steady-state if it had been administered for ≥7 days to allow for saturation of first-pass metabolism and the establishment of metabolic autoinduction.

### Data synthesis

In many of the studies, more than one group of participants was compared, e.g. HIV-positive and HIV-negative participants.^27^ In others, more than one treatment was compared, e.g. in a crossover trial comparing separate drug formulations with fixed-dose combinations.^28^ These groups were analysed in the same way that data were presented in the papers; that is, separate study arms were analysed separately rather than mean values being calculated for each study. This meant that some studies contributed two or more sets of PK parameters to the meta-analysis. To enable comparison of PK parameters across all studies, data were collected as means and standard deviations. Where summary statistics were not published in this format, authors were contacted to request that they share either raw data or results of an NCA of their data. If data were summarized as median and range or IQR and raw data or NCA results were unobtainable from the authors, we estimated the mean and standard deviation from the summary statistics provided using previously described methods.^29^

As the *C*_max_ of rifampicin occurs around 2 h after ingestion and half-life is of the order of 2.5–4 h,^30^ concentrations remaining in plasma after 24 h from ingestion will be negligible. This was supported by the lack of a statistically significant difference between the estimates of AUC produced from the 0–24 h time interval and the 0–48 h time interval and those calculated from the 0–infinity (∞) interval. The AUC_0–24_, AUC_0–48_ and AUC_0–∞_ results were therefore combined into a single measure of AUC and only these estimates were included in the final analysis to minimize design-related heterogeneity. Hereafter, any reference to AUC refers to the combined AUC_0–24_, AUC_0–48_ and AUC_0–∞_ estimates. Although rifampicin is 80%–90% protein bound and the active portion is believed to be unbound drug, studies reported total drug PK parameters; this analysis used the same.^15^^,^^30^

### Summary measures

Data were analysed in Microsoft Excel version 15.28 (Microsoft 2016) and using the metafor package in R version 3.3.1.^31^ The main objective of the analysis was to collate and summarize available data on the PK parameters of rifampicin derived from subjects taking WHO-recommended dosages. The focus of the meta-analysis was therefore on the 8–12 mg/kg dosing bracket. A linear model was used to incorporate the following variables: HIV status (positive or negative); TB status (positive or negative); combination therapy [limited to patients taking rifampicin monotherapy versus those taking combination therapy with isoniazid, pyrazinamide and ethambutol (RHZE)]; intermittent dosing; diabetes status; and treatment duration. A restricted maximum likelihood mixed-effects model was used to perform a meta-analysis of *C*_max_ and AUC estimates, with application of the DerSimonian–Laird estimator of residual heterogeneity. This approach fits a random-effects model. Standard errors of the study-specific estimates are adjusted to incorporate a measure of the heterogeneity among the effects of independent variables observed in different studies.^32^ The degree to which demographic and clinical variables accounted for inter-study heterogeneity was assessed using meta-regression. Heterogeneity of PK estimates overall and within subgroups was assessed by estimation of the *I*^2^ statistic and visual inspection of forest plots.

A second objective was to explore the effect of higher-than-recommended doses of rifampicin on drug exposure. The >12 mg/kg group of studies was split into more specific dosing subgroups and the mean and standard error derived from meta-analysis in standard weight-based dosing categories was compared with the summary statistics extracted from studies of higher rifampicin dosages. As the number of studies at higher dosages was small, we were unable to incorporate dose escalation as a variable in the meta-regression, so graphical comparison of summary statistics from studies at standard and higher dosages was performed instead.

## Results

The search retrieved 3075 titles, of which 70 studies were deemed eligible, containing 179 distinct study arms (Figure S1, available as Supplementary data at *JAC* Online). The characteristics of the studies are summarized in Table S1. The cohorts contained a total of 3477 study participants. HPLC was used to measure rifampicin levels in 66 of the 70 studies. The remaining studies used spectrophotometry^33–35^ or a plate diffusion assay.^36^ These three studies were retained in the meta-analysis because their exclusion did not significantly impact overall PK parameter estimates.

**Table 1.** Univariate analysis of variables influencing estimated rifampicin AUC

Variable and category	Number of study arms	Number of patients	AUC estimate (mg·h/L)	95% CI	SEM	*P*
Duration of therapy						
single dose	58	1053	72.56	66.39–78.74	2.60	<0.0001
steady-state dosing (>1 week)	34	846	38.73	33.82–42.67	4.33	
HIV status						
HIV negative	14	236	56.66	47.37–65.96	4.08	
HIV positive	9	126	37.16	27.08–47.23	6.56	0.003^a^
mixed HIV population	14	569	41.36	34.82–47.90	5.77	0.005^a^
TB status						
TB patients	36	947	46.14	39.39–52.89	5.29	<0.0001
healthy volunteers	56	952	69.41	62.17–76.66	3.31	
Drug combination						
rifampicin monotherapy	11	122	63.21	54.53–71.89	4.43	0.0478
RHZE	39	842	51.70	40.29–63.11	5.82	
Diabetes status						
no diabetes	12	227	84.56	73.70–95.42	5.54	0.44
diabetes	2	42	73.17	44.46–101.88	14.65	
Dosing frequency						
daily dosing	87	1617	61.52	55.62–67.42	3.01	0.35
intermittent dosing	3	189	46.01	13.69–78.33	16.49	

Univariate analysis indicated significant differences in estimated AUC depending on treatment duration, HIV status, TB status and combination therapy.

Steady-state refers to dosing for ≥7 days to allow for saturation of first-pass metabolism and the establishment of metabolic autoinduction.

*P* values indicate significance of difference between pooled AUC estimates within each study variable.

a *P* value for difference from HIV-negative population.

By far the most common weight-based dosing category in the included studies was 8–12 mg/kg (118 of 163 study arms for which dosing information was extracted, 72%), in line with WHO rifampicin dosing guidelines. Unless explicitly stated, results presented hereafter pertain to those studies in which patients received this recommended dose.

### C_max_ data were highly heterogeneous and influenced by treatment duration

*C*_max_ was highly heterogeneous between studies, with an *I*^2^ statistic of 95.36% (95% CI 95.13%–97.15%). Meta-regression of *C*_max_ estimates with a multivariate model including all variables found two modifiers to have a statistically significant impact on *C*_max_: duration of treatment and TB status. The effect on inter-study variability was minor, however: *I*^2^ = 91.36% (95% CI 90.50%–94.77%) after meta-regression. The population summary estimates for *C*_max_ after univariate analysis were 11.51 mg/L (SEM 0.38) after single dosing and 7.04 mg/L (SEM 0.58) after steady-state dosing (*P *=* *0.001) (Figure S2). In multivariate analysis, the difference in *C*_max_ estimate according to dosing duration was upheld. Single dosing (*n *=* *1139 in 66 study arms) resulted in an adjusted mean *C*_max_ of 8.98 mg/L (SEM 1.34) and steady-state dosing (*n *=* *904 in 42 study arms) resulted in an adjusted *C*_max_ of 5.79 mg/L (SEM 0.90) (*P *=* *0.001). The adjusted summary estimate of *C*_max_ for healthy volunteers (*n *=* *946 in 60 study arms) as compared with TB patients (*n *=* *1075 in 46 study arms) was 8.98 mg/L (SEM 1.34) in healthy volunteers and 6.39 mg/L (SEM 0.85) in TB patients (*P *=* *0.01). Notably, the majority of healthy volunteer cohorts were studied after a single dose of rifampicin (109/120 healthy volunteer cohorts, 91%) and most TB patients were studied after steady-state dosing (53/63 TB patient cohorts, 84%). When multivariate analysis was limited to subjects dosed at steady-state, TB status had a negligible and non-significant modifying effect on *C*_max_: healthy volunteers 7.08 mg/L (SEM 1.21); TB patients 7.04 mg/L (SEM 1.28) (*P *=* *0.98). No other modifying variables had a significant impact on the adjusted *C*_max_ estimate (Table S2).

**Table 2.** Meta-regression of variables influencing estimated rifampicin AUC

Variable and category	Adjusted AUC estimate (mg·h/L)	95% CI	SEM	*P*
Duration of therapy				
single dose	56.26	29.01–83.50	13.90	<0.0001
steady-state dosing (>1 week)	20.94	8.28–33.60	6.49	<0.0001
HIV status				
HIV negative	53.16	41.63–64.68	5.85	0.60
HIV positive	48.13	33.26–63.61	7.74	0.31
mixed HIV population	54.53	37.08–71.98	8.90	0.85
TB status				
TB patients	56.26	43.22–69.29	6.65	0.10
healthy volunteers	67.09	54.11–80.07	6.62	0.10
Drug combination				
rifampicin monotherapy	87.71	59.48–113.93	13.89	0.72
RHZE	72.19	50.91–101.47	12.90	0.67
Diabetes status				
no diabetes	109.97	61.03–158.91	24.97	0.03
diabetes	113.30	59.03–167.55	27.68	0.04
Dosing frequency				
daily dosing	54.94	24.42–85.46	15.57	0.93
intermittent dosing	39.02	17.01–60.95	11.18	0.12

Meta-regression of all available variables found that treatment duration alone had a substantial and significant impact on estimated rifampicin AUC.

Steady-state refers to dosing for ≥7 days to allow for saturation of first-pass metabolism and the establishment of metabolic autoinduction.

*P* values indicate significance of difference between pooled AUC estimates and overall population estimate.

### Only treatment duration had a consistently significant impact on AUC in univariate analysis

**Figure 1.**
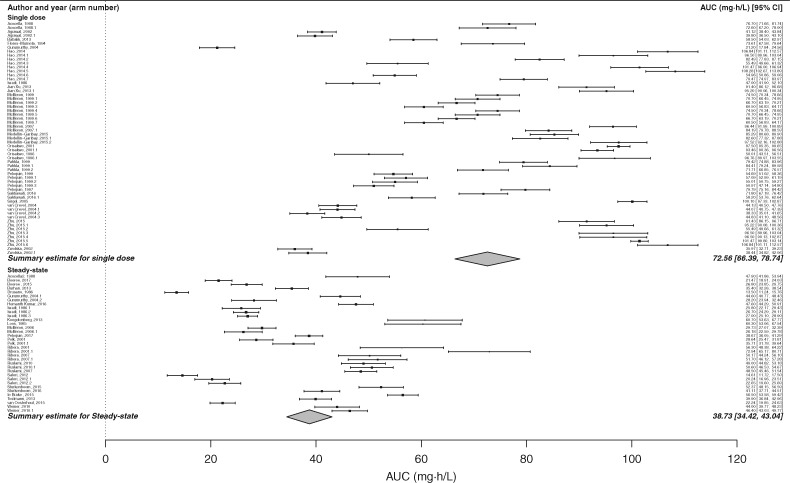
Forest plot displaying estimated rifampicin AUC after univariate analysis according to dosing duration. In univariate analysis, the effect of steady-state dosing was to approximately halve the estimated rifampicin AUC (*P *<* *0.0001).

**Figure 2.**
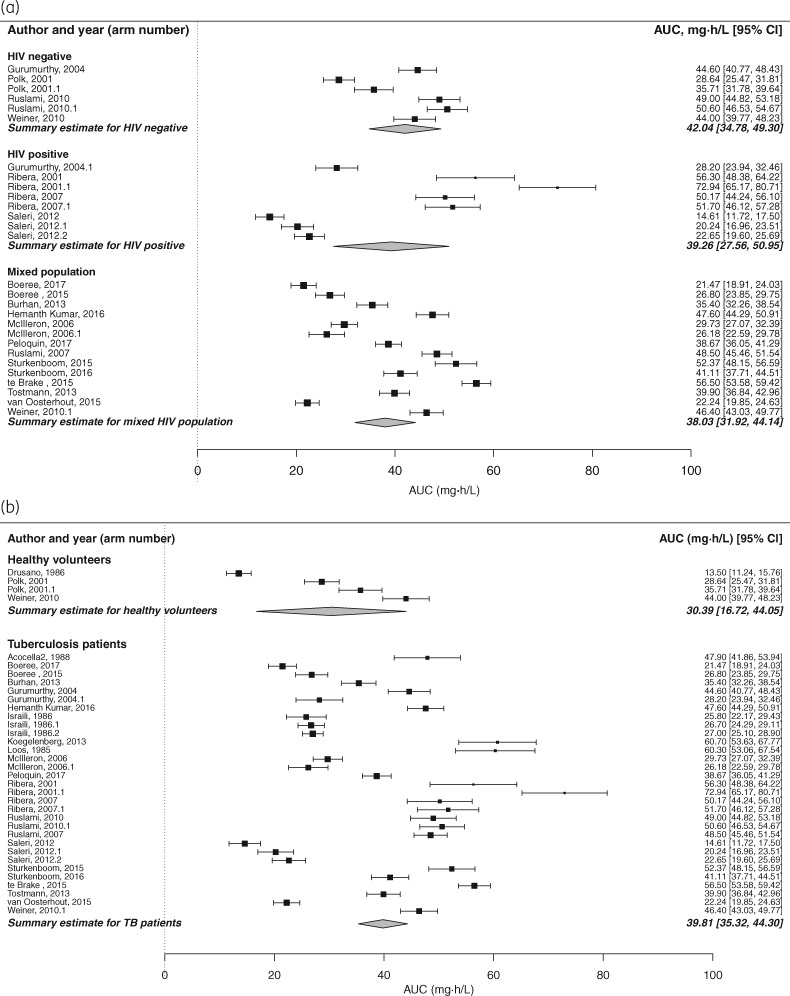
(a) Forest plot displaying estimated rifampicin AUC after univariate analysis according to HIV status; data are limited to steady-state dosing. Once data were limited to steady-state dosing, HIV status no longer had a significant impact on rifampicin AUC estimate. *P* values for comparison were >0.05. (b) Forest plot displaying estimated rifampicin AUC after univariate analysis according to TB status; data are limited to steady-state dosing. Once data were limited to steady-state dosing, TB status no longer had a significant impact on the rifampicin AUC estimate. *P* value for comparison was >0.05.

In keeping with the findings in relation to the *C*_max_ estimate, inter-study variability in the AUC estimate was extreme, with an *I*^2^ statistic of 99.53% (95% CI 99.28%–99.60%) in the meta-analysis before inclusion of modifying variables. In univariate analysis, the effect of steady-state dosing was to approximately halve the mean AUC estimate, from 72.56 (SEM 2.60) to 38.73 mg·h/L (SEM 4.33) (*P *<* *0.0001) (Table 1 and Figure 1). Univariate analysis indicated significant associations between the AUC estimate and three additional covariates: HIV status, TB status and whether rifampicin was dosed in monotherapy or in combination (Table 1). However, steady-state dosing was disproportionately represented compared with single dosing in both HIV-positive patients and TB patients (100% and 82% of HIV-positive and TB patients, respectively, were studied at steady-state). Once these analyses were repeated with data limited to steady-state dosing, neither HIV status nor TB status had a significant impact on the AUC estimate (Figure 2a and b). Similarly, when the analysis was limited to those who underwent steady-state dosing, combination therapy made no significant difference to the AUC estimate: AUC 39.54 (SEM 3.83) versus 36.73 mg·h/L (SEM 4.88) for rifampicin monotherapy versus RHZE combination therapy (*P *=* *0.57).

### Significance of effect of treatment duration on AUC was upheld in meta-regression, but vast heterogeneity remained

When all modifying variables were incorporated into a mixed-effects meta-regression model, the impact on inter-study heterogeneity was negligible (*I*^2^ = 98.69%, 95% CI 98.38%–99.14%). Only treatment duration had a significant impact on AUC: adjusted AUC 56.26 mg·h/L (SEM 13.90) after a single dose and 20.94 mg·h/L (SEM 6.49) after steady-state dosing (Table 2). After multivariate meta-regression analysis, combination therapy with RHZE no longer had a significant impact on AUC. A diagnosis of diabetes had a negligible, although statistically significant, modifying effect on the AUC estimate (Table 2).

### Current rifampicin dosages for TB are unlikely to be sufficient for PK/PD target attainment

**Figure 3.**
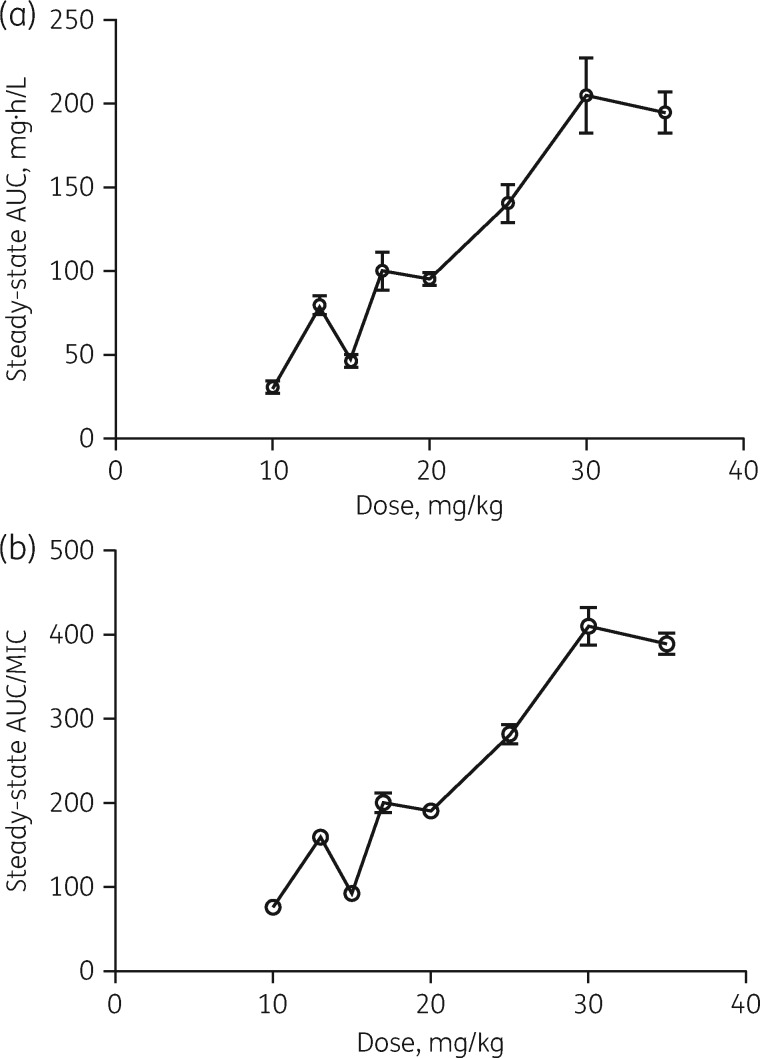
(a) Impact of increasing dose on rifampicin AUC. With increasing dose, there appears to be a greater than proportional increase in AUC. Error bars show SEM. Data are displayed in Table 3. (b) Impact of increasing dose on rifampicin AUC/MIC. Taking the ECOFF MIC of 0.5 mg/L, available data indicate that a rifampicin dose of ≥25 mg/kg is required to attain the PK/PD target associated with a 1 log cfu reduction (an AUC/MIC of 271).

There appeared to be a slightly greater than proportional increase in AUC with increasing dosage (Table 3 and Figure 3a), although additional data from ongoing trials will help to clarify this. In seeking to relate these reported drug exposures to measures of clinical outcome, we used published PK/PD indices associated with efficacy in murine studies^21^ and MIC data from human clinical WT *M. tuberculosis* isolates.^37^ These murine studies report that an AUC/MIC of 271 is required for a 1 log cfu reduction *in vivo*.^21^ The rifampicin WT MIC distribution ranges from 0.03 to 0.5 mg/L, with a median of 0.25 mg/L and proposed epidemiological cut-off value (ECOFF) of 0.5 mg/L.^37^ Taking the median WT MIC of 0.25 mg/L, doses of 13 mg/kg appear sufficient to achieve the AUC/MIC target of 271. Taking the ECOFF MIC of 0.5 mg/L, however, available data indicate that a rifampicin dose of ≥25 mg/kg is required to attain this PK/PD target associated with a 1 log cfu reduction (Figure 3b).

**Table 3.** Rifampicin AUC at steady-state: meta-analysed standard dose compared with higher dosages

Rifampicin dose (mg/kg)	Number of subjects	Mean AUC (mg·h/L)	SEM	References
8–12	846	38.2	4.3	^a^
13	23	79.7	5.4	^16^
15	55	46.4	3.4	^49^
17	11	100.1	11.0	^50^
20	113	95.2	3.8	^23^^,^^49–51^
25	15	140.5	11.2	^23^
30	15	204.8	22.6	^23^
35	35	194.6	12.3	^23^^,^^51^

With increasing dose, there is a greater than proportional increase in AUC. Data are displayed in Figure 3(a).

Steady-state refers to dosing for ≥7 days to allow for saturation of first-pass metabolism and the establishment of metabolic autoinduction.

a All references in meta-analysis (see Table S1).

## Discussion

This meta-analysis, to our knowledge the most comprehensive to have been conducted on rifampicin PK, has demonstrated vast inter-study heterogeneity in PK parameter estimates. Having collated data collected globally, spanning 35 years and with the inclusion of HIV status, TB status, combination therapy, intermittent dosing, diabetes status and treatment duration as modifying variables, we have been unable to explain this heterogeneity. The vast heterogeneity within and between studies has made it impossible to assess the degree to which physiological differences between individual patients impacts upon rifampicin PK or PK variability, as has been reported with other antimicrobials.^38^^,^^39^

The summary estimates of *C*_max_ and AUC will serve as useful reference points for clinicians and academics concerned with the dosing of rifampicin for TB. At standard, WHO-recommended doses, mean rifampicin *C*_max_ and AUC are both significantly reduced in patients dosed at steady-state: *C*_max_ 8.98 versus 5.79 mg/L and AUC 72.56 versus 38.73 mg·h/L after a single dose and steady-state dosing, respectively. These decreases in PK parameters are expected due to extensive, saturable first-pass metabolism and well-characterized autoinduction of metabolism, resulting in enhanced clearance after repeated doses.^30^^,^^40^^,^^41^ Whilst there was a trend towards HIV positivity being associated with lower rifampicin AUC, this did not hold up in meta-regression analysis, which may explain the conflicting results of previous investigations into the effect of HIV positivity on rifampicin exposure.^5^^,^^27^^,^^42–44^ The case of AUC in TB patients versus healthy volunteers was similar in that the significance of the association was lost in meta-regression analysis. 

With increasing dose, there is a greater than proportional increase in AUC. This is encouraging for the community that is seeking to increase rifampicin exposure. Taking 38.73 mg·h/L as the mean rifampicin AUC at steady-state dosing of 8–12 mg/kg and the ECOFF MIC of 0.5 mg/L^37^ gives an AUC/MIC ratio of 77, far below the optimal PK/PD index suggested by Jayaram *et al.*^21^ from murine data (prior to reference). Taking the MIC value from the very lower end of the WT range (0.03 mg/L) gives a ratio of 1291. The discrepancy between these ratios may explain in part why some patients develop rifampicin resistance on currently recommended doses while others are successfully treated with the same dose. The PK variability demonstrated herein is likely also to contribute to this phenomenon. Of note, this PK/PD index indicates the potency of a single drug used in isolation and does not reflect the efficacy of rifampicin used in clinical settings and in combination with other agents. There are also likely to be microbiological and host immune factors that influence treatment success. Our calculations nevertheless highlight the inadequacy of current rifampicin doses and the need for these to increase. 

This analysis is limited by the fact that many studies summarized their results as median and range or IQR and, as stated, where raw data could not be obtained from authors of those studies means and standard errors were estimated using a previously described method.^29^ This may have introduced inaccuracies. Our categorization of studies according to weight-based dosing was necessarily crude and in some cases based on the average weight of the study population in question. In addition, we were not able to consider the impact of covariates that were not consistently measured on heterogeneity in PK estimates. These included co-medications and associated drug–drug interactions, specific formulations of rifampicin that have been demonstrated to exhibit altered PK,^33^^,^^45^^,^^46^ and patient ethnicity.

We acknowledge that the heterogeneity amongst the included studies, likely caused in part by these and other design and reporting factors, is extreme. Nevertheless, we believe that our largely descriptive analysis has value in highlighting the importance of these factors, in addition to the widely recognized role of inter-individual variability, in terms of their impact on the PK of rifampicin.^47^^,^^48^ The extreme residual inter-study variability not accounted for by our meta-regression analysis may thus represent significant true biological variability between study populations, which should be further explored. In addition, the degree of PK variability that is attributable to protein-bound versus unbound rifampicin is not known. Future studies that directly assess these factors would be valuable, as would studies that employ mathematical PK models to quantify rifampicin PK variability. Monte Carlo simulation of rifampicin exposure based upon the AUC distributions presented in this meta-analysis would enable exploration of various dosing regimens. If these simulations could incorporate predictions of toxicity and drug resistance, they would support risk reduction of novel regimens before they enter clinical use.

This meta-analysis has collated and quantitatively summarized the existing literature on the PK of rifampicin, which is believed to be the key driver of PD and ultimately treatment outcome. It provides an important point of reference for understanding rifampicin efficacy at current dosages as exploration of higher dosages continues.

## Supplementary Material

Supplementary Data

## References

[1] Zumla A, Nahid P, Cole ST. Advances in the development of new tuberculosis drugs and treatment regimens. Nat Rev Drug Discov 2013; 12: 388–404. 23629506 10.1038/nrd4001

[2] WHO, ‘StopTB’ Initiative. *Guidelines for Treatment of Tuberculosis, Fourth Edition* http://www.who.int/tb/publications/2010/9789241547833/en/.

[3] van Ingen J, Aarnoutse RE, Donald PR et al Why do we use 600 mg of rifampicin in tuberculosis treatment? Clin Infect Dis 2011; 52: e194–9. 21467012 10.1093/cid/cir184

[4] Peloquin C. Therapeutic drug monitoring: principles and applications in mycobacterial infections. Drug Therapy 1992; 22: 31–6.

[5] Peloquin CA, Nitta AT, Burman WJ et al Low antituberculosis drug concentrations in patients with AIDS. Ann Pharmacother 1996; 30: 919–25. 8876848 10.1177/106002809603000901

[6] Magis-Escurra C, van den Boogaard J, Ijdema D et al Therapeutic drug monitoring in the treatment of tuberculosis patients. Pulm Pharmacol Ther 2012; 25: 83–6. 22179055 10.1016/j.pupt.2011.12.001

[7] Babalik A, Mannix S, Francis D et al Therapeutic drug monitoring in the treatment of active tuberculosis. Can Respir J 2011; 18: 225–9. 22059181 10.1155/2011/307150 PMC3205104

[8] Holland DP, Hamilton CD, Weintrob AC et al Therapeutic drug monitoring of antimycobacterial drugs in patients with both tuberculosis and advanced human immunodeficiency virus infection. Pharmacotherapy 2009; 29: 503–10. 19397460 10.1592/phco.29.5.503

[9] Tappero JW, Bradford WZ, Agerton TB et al Serum concentrations of antimycobacterial drugs in patients with pulmonary tuberculosis in Botswana. Clin Infect Dis 2005; 41: 461–9. 16028152 10.1086/431984

[10] Chideya S, Winston CA, Peloquin CA et al Isoniazid, rifampin, ethambutol, and pyrazinamide pharmacokinetics and treatment outcomes among a predominantly HIV-infected cohort of adults with tuberculosis from Botswana. Clin Infect Dis 2009; 48: 1685–94. 19432554 10.1086/599040 PMC3762461

[11] Hemanth Kumar AK, Kannan T, Chandrasekaran V et al Pharmacokinetics of thrice-weekly rifampicin, isoniazid and pyrazinamide in adult tuberculosis patients in India. Int J Tuberc Lung Dis 2016; 20: 1236–41. 27510252 10.5588/ijtld.16.0048

[12] Heysell SK, Moore JL, Keller SJ et al Therapeutic drug monitoring for slow response to tuberculosis treatment in a state control program, Virginia, USA. Emerg Infect Dis 2010; 16: 1546–53. 20875279 10.3201/eid1610.100374 PMC3294393

[13] Chigutsa E, Pasipanodya JG, Visser ME et al Impact of nonlinear interactions of pharmacokinetics and MICs on sputum bacillary kill rates as a marker of sterilizing effect in tuberculosis. Antimicrob Agents Chemother 2015; 59: 38–45. 25313213 10.1128/AAC.03931-14 PMC4291375

[14] Ji B, Truffot-Pernot C, Lacroix C et al Effectiveness of rifampin, rifabutin, and rifapentine for preventive therapy of tuberculosis in mice. Am Rev Respir Dis 1993; 148: 1541–6. 8256897 10.1164/ajrccm/148.6_Pt_1.1541

[15] Alsultan A, Peloquin CA. Therapeutic drug monitoring in the treatment of tuberculosis: an update. Drugs 2014; 74: 839–54. 24846578 10.1007/s40265-014-0222-8

[16] Ruslami R, Nijland HM, Alisjahbana B et al Pharmacokinetics and tolerability of a higher rifampin dose versus the standard dose in pulmonary tuberculosis patients. Antimicrob Agents Chemother 2007; 51: 2546–51. 17452486 10.1128/AAC.01550-06 PMC1913243

[17] Diacon AH, Patientia RF, Venter A et al Early bactericidal activity of high-dose rifampin in patients with pulmonary tuberculosis evidenced by positive sputum smears. Antimicrob Agents Chemother 2007; 51: 2994–6. 17517849 10.1128/AAC.01474-06 PMC1932511

[18] Davies GR, Nuermberger EL. Pharmacokinetics and pharmacodynamics in the development of anti-tuberculosis drugs. Tuberculosis (Edinb) 2008; 88 Suppl 1: S65–74. 18762154 10.1016/S1472-9792(08)70037-4

[19] Mitnick CD, McGee B, Peloquin CA. Tuberculosis pharmacotherapy: strategies to optimize patient care. Expert Opin Pharmacother 2009; 10: 381–401. 19191677 10.1517/14656560802694564 PMC2674232

[20] Gumbo T, Louie A, Deziel MR et al Concentration-dependent *Mycobacterium tuberculosis* killing and prevention of resistance by rifampin. Antimicrob Agents Chemother 2007; 51: 3781–8. 17724157 10.1128/AAC.01533-06 PMC2151424

[21] Jayaram R, Gaonkar S, Kaur P et al Pharmacokinetics-pharmacodynamics of rifampin in an aerosol infection model of tuberculosis. Antimicrob Agents Chemother 2003; 47: 2118–24. 12821456 10.1128/AAC.47.7.2118-2124.2003 PMC161844

[22] de Steenwinkel JE, Aarnoutse RE, de Knegt GJ et al Optimization of the rifampin dosage to improve the therapeutic efficacy in tuberculosis treatment using a murine model. Am J Respir Crit Care Med 2013; 187: 1127–34. 23525933 10.1164/rccm.201207-1210OC

[23] Boeree MJ, Diacon AH, Dawson R et al A dose-ranging trial to optimize the dose of rifampin in the treatment of tuberculosis. Am J Respir Crit Care Med 2015; 191: 1058–65. 25654354 10.1164/rccm.201407-1264OC

[24] Pasipanodya JG, McIlleron H, Burger A et al Serum drug concentrations predictive of pulmonary tuberculosis outcomes. J Infect Dis 2013; 208: 1464–73. 23901086 10.1093/infdis/jit352 PMC3789573

[25] Moher D, Liberati A, Tetzlaff J et al Preferred reporting items for systematic reviews and meta-analyses: the PRISMA statement. PLoS Med 2009; 151: 264–9, w64. 10.7326/0003-4819-151-4-200908180-00135 19622511

[26] Roberts JA, Taccone FS, Lipman J. Understanding PK/PD. Intensive Care Med 2016; 42: 1797–800. 26334756 10.1007/s00134-015-4032-6

[27] Choudhri SH, Hawken M, Gathua S et al Pharmacokinetics of antimycobacterial drugs in patients with tuberculosis, AIDS, and diarrhea. Clin Infect Dis 1997; 25: 104–11. 9243044 10.1086/514513

[28] Agrawal S, Singh I, Kaur KJ et al Bioequivalence assessment of rifampicin, isoniazid and pyrazinamide in a fixed dose combination of rifampicin, isoniazid, pyrazinamide and ethambutol vs. separate formulations. Int J Clin Pharmacol Ther 2002; 40: 474–81. 12395981 10.5414/cpp40474

[29] Wan X, Wang W, Liu J et al Estimating the sample mean and standard deviation from the sample size, median, range and/or interquartile range. BMC Med Res Methodol 2014; 14: 135. 25524443 10.1186/1471-2288-14-135 PMC4383202

[30] Acocella G. Clinical pharmacokinetics of rifampicin. Clin Pharmacokinet 1978; 3: 108–27. 346286 10.2165/00003088-197803020-00002

[31] Viechtbauer W. Conducting meta-analyses in R with the metafor package. J Stat Softw 2010; 36: 1–48.

[32] DerSimonian R, Laird N. Meta-analysis in clinical trials. Control Clin Trials 1986; 7: 177–88. 3802833 10.1016/0197-2456(86)90046-2

[33] Garg SK, Chakrabarti A, Panigrahi D et al Comparative bioavailability and in-vitro antimicrobial activity of two different brands of rifampicin. Eur J Drug Metab Pharmacokinet 1991; 16: 223–9. 1814740 10.1007/BF03189964

[34] Orisakwe OE, Ofoefule SI. Plasma and saliva concentrations of rifampicin in man after oral administration. Tokai J Exp Clin Med 1996; 21: 45–9. 9239804

[35] Orisakwe OE, Agbasi PU, Afonne OJ et al Rifampicin pharmacokinetics with and without ciprofloxacin. Am J Ther 2001; 8: 151–3. 11344382 10.1097/00045391-200105000-00003

[36] Potkar C, Gogtay N, Gokhale P et al Phase I pharmacokinetic study of a new 3-azinomethyl-rifamycin (rifametane) as compared to rifampicin. Chemotherapy 1999; 45: 147–53. 10224335 10.1159/000007176

[37] Schon T, Jureen P, Giske CG et al Evaluation of wild-type MIC distributions as a tool for determination of clinical breakpoints for *Mycobacterium tuberculosis*. J Antimicrob Chemother 2009; 64: 786–93. 19633001 10.1093/jac/dkp262

[38] Roberts JA, Abdul-Aziz MH, Lipman J et al Individualised antibiotic dosing for patients who are critically ill: challenges and potential solutions. Lancet Infect Dis 2014; 14: 498–509. 24768475 10.1016/S1473-3099(14)70036-2 PMC4181663

[39] Alobaid AS, Wallis SC, Jarrett P et al Effect of obesity on the population pharmacokinetics of fluconazole in critically ill patients. Antimicrob Agents Chemother 2016; 60: 6550–7. 27550344 10.1128/AAC.01088-16 PMC5075058

[40] Chen J, Raymond K. Roles of rifampicin in drug-drug interactions: underlying molecular mechanisms involving the nuclear pregnane X receptor. Ann Clin Microbiol Antimicrob 2006; 5: 3. 16480505 10.1186/1476-0711-5-3 PMC1395332

[41] Loos U, Musch E, Jensen JC et al Pharmacokinetics of oral and intravenous rifampicin during chronic administration. Klin Wochenschr 1985; 63: 1205–11. 4087830 10.1007/BF01733779

[42] Schaaf HS, Willemse M, Cilliers K et al Rifampin pharmacokinetics in children, with and without human immunodeficiency virus infection, hospitalized for the management of severe forms of tuberculosis. BMC Med 2009; 7: 19. 19386087 10.1186/1741-7015-7-19 PMC2679060

[43] Ahmed R, Cooper R, Foisy M et al Factors associated with reduced antituberculous serum drug concentrations in patients with HIV-TB coinfection. J Int Assoc Physicians AIDS Care (Chic) 2012; 11: 273–6. 22875581 10.1177/1545109712454454

[44] Gurumurthy P, Ramachandran G, Hemanth Kumar AK et al Decreased bioavailability of rifampin and other antituberculosis drugs in patients with advanced human immunodeficiency virus disease. Antimicrob Agents Chemother 2004; 48: 4473–5. 15504887 10.1128/AAC.48.11.4473-4475.2004 PMC525439

[45] McIlleron H, Wash P, Burger A et al Widespread distribution of a single drug rifampicin formulation of inferior bioavailability in South Africa. Int J Tuberc Lung Dis 2002; 6: 356–61. 11936746

[46] Nyazema NZ, Rabvukwa P, Gumbo J et al Bioavailability of rifampicin in a separate formulation and fixed dose combination with isoniazid NIH: a case for a fixed dose combination (FDC) for the treatment of tuberculosis. Cent Afr J Med 1999; 45: 141–4. 10695183 10.4314/cajm.v45i6.8472

[47] Schipani A, Pertinez H, Mlota R et al A simultaneous population pharmacokinetic analysis of rifampicin in Malawian adults and children. Br J Clin Pharmacol 2016; 81: 679–87. 26613187 10.1111/bcp.12848 PMC4799933

[48] Verbeeck RK, Günther G, Kibuule D et al Optimizing treatment outcome of first-line anti-tuberculosis drugs: the role of therapeutic drug monitoring. Eur J Clin Pharmacol 2016; 72: 905–16. 27305904 10.1007/s00228-016-2083-4

[49] Peloquin CA, Velásquez GE, Lecca L et al Pharmacokinetic evidence from the HIRIF trial to support increased doses of rifampin for tuberculosis. Antimicrob Agents Chemother 2017; 61: e00038–17. 28559269 10.1128/AAC.00038-17 PMC5527578

[50] Yunivita V, Dian S, Ganiem AR et al Pharmacokinetics and safety/tolerability of higher oral and intravenous doses of rifampicin in adult tuberculous meningitis patients. Int J Antimicrob Agents 2016; 48: 415–21. 27526979 10.1016/j.ijantimicag.2016.06.016

[51] Boeree MJ, Heinrich N, Aarnoutse R et al High-dose rifampicin, moxifloxacin, and SQ109 for treating tuberculosis: a multi-arm, multi-stage randomised controlled trial. Lancet Infect Dis 2017; 17: 39–49. 28100438 10.1016/S1473-3099(16)30274-2 PMC5159618

