# Supplementary material for: Pharmacokinetics of rifampicin in adult TB patients and healthy volunteers: a systematic review and meta-analysis
Source: J Antimicrob Chemother. 2018 Apr 26;73(9):2305–13. doi: 10.1093/jac/dky152 (PMC6105874; doi:10.1093/jac/dky152)
Supplement: Supplementary Data [file dky152_supplementary_data.docx]

**Supplementary data**

**Table S1.** Characteristics of studies included in meta-analysis

| Author & Year | Study arms | Regimen | Number of subjects in study arm | Dose of rifampicin | Weight-based dosing category* | Schedule | Study population | Dosing frequency | Formulation | HIV Status | Diabetes status | AUC timeframe |
| --- | --- | --- | --- | --- | --- | --- | --- | --- | --- | --- | --- | --- |
| Acocella 1988^1^ | 2 | RHZ | 10 | 600mg | B | Single dose | Healthy volunteers | Regular | Separate formulation | NR | NR | 0-24 |
|  |  | RHZ | 10 |  |  |  |  |  | FDC |  |  |  |
| Acocella 1993^2^ | 2 | RHZ | 6 | 600mg | NR | Single dose | Healthy volunteers | Regular | Separate formulation | NR | NR | 0-infinity |
|  |  | RHZ | 6 |  |  |  |  |  | FDC |  |  |  |
| Acocella2 1988^3^ | 2 | RHZ | 13 | Mean dose 11.5mg/kg | B | Steady state | TB patients | Regular | FDC | NR | NR | 0-12 |
|  |  | RHZ | 13 |  |  |  |  |  | FDC |  |  | 0-infinity |
| Agrawal 2002^4^ | 2 | RHZE | 14 | 450mg | B | Single dose | Healthy volunteers | Regular | FDC | NR | NR | 0-24 |
|  |  | RHZE | 14 |  |  |  |  |  | Separate formulation |  |  |  |
| Agrawal 2004^5^ | 2 | RHZE | 22 | 600mg | NR | Single dose | Healthy volunteers | Regular | FDC | NR | NR | 0-24 |
|  |  | RHZE | 22 |  |  |  |  |  | Separate formulation |  |  |  |
| Agrawal 2001^6^ | 2 | RHZE | 14 | 450mg | NR | Single dose | Healthy volunteers | Regular | FDC | NR | NR | 0-infinity |
|  |  | RHZE | 14 |  |  |  |  |  | Separate formulation |  |  |  |
| Agrawal2 2002^7^ | 2 | RHZE | 14 | 450mg | NR | Single dose | Healthy volunteers | Regular | FDC | NR | NR | 0-24 |
|  |  | RHZE | 14 |  |  |  |  |  | Separate formulation |  |  |  |
| Ahn 2003^8^ | 1 | RHZ | 9 | 450mg | A | Single dose | Healthy volunteers | Regular | Separate formulation | NR | NR | 0-infinity |
| Ashokraj 2005^9^ | 2 | RHE | 20 | 450mg | NR | Single dose | Healthy volunteers | Regular | FDC | Negative | NR | 0-24 |
|  |  | RHE | 20 |  |  |  |  |  |  |  |  |  |
| Avachat 2011^10^ | 1 | RH | 8 | 300mg | NR | Single dose | Healthy volunteers | Regular | Separate formulation | NR | NR | 0-infinity |
| Babalik 2013^11^ | 1 | RHZE | 21 | 600mg | B | Single dose | TB TB patients | Regular | Separate formulation | Negative | NR | 0-24 |
| Boeree 2015^12^ | 5 | RHZE | 8 | 10mg/kg | B | Steady state | TB patients | Regular | NR | Mixed population | NR | 0-24 |
|  |  | RHZE | 15 | 20mg/kg | C | Steady state | TB patients | Regular | NR | Mixed population | NR | 0-24 |
|  |  | RHZE | 15 | 25mg/kg |  |  |  |  |  |  |  |  |
|  |  | RHZE | 15 | 30mg/kg |  |  |  |  |  |  |  |  |
|  |  | RHZE | 15 | 35mg/kg |  |  |  |  |  |  |  |  |
| Boeree 2017 ^13^ | 5 | RHZE | 20 | 35mg/kg | C | Steady state | TB patients | Regular | NR | Mixed population | NR |  |
|  |  | RM | 19 | 20mg/kg | C |  |  |  |  |  |  |  |
|  |  | RS | 20 | 20mg/kg | C |  |  |  |  |  |  |  |
|  |  | RS | 20 | 10mg/kg | B |  |  |  |  |  |  |  |
|  |  | RHZE | 19 | 10mg/kg | B |  |  |  |  |  |  |  |
| Burhan 2013^14^ | 1 | RHZ | 9 | 30-39kg 300mg; 40-54kg 450mg; 55-70kg 600mg, >70kg 750mg | B | Steady state | TB patients | Regular | FDC | Mixed population | Mixed population | 0-24 |
| Chik 2010^15^ | 2 | R | 14 | 300mg | A | Single dose | Healthy volunteers | Regular | Separate formulation | Negative | NR | 0-24 |
|  |  | R | 14 |  |  |  |  |  |  |  |  |  |
| Choudhri 1997^16^ | 4 | RHZ | 29 | 600mg | B | Steady state | TB patients | Regular | Separate formulation | Positive | NR | 0-12 |
|  |  | RHZ | 29 |  |  |  |  |  |  | Negative |  |  |
|  |  | RHZ | 29 |  |  |  |  |  |  | NR |  |  |
|  |  | RHZ | 29 |  |  |  |  |  |  |  |  |  |
| Drusano 1986^17^ | 1 | R | 10 | 600mg | B | Steady state | Healthy volunteers | Regular | Separate formulation | NR | NR | 0-24 |
| Flores-Murrieta 1994^18^ | 1 | R | 8 | 600mg | B | Single dose | Healthy volunteers | Regular | Separate formulation | NR | NR | 0-24 |
| Garg 1991^19^ | 8 | R | 8 | 300mg | A | Single dose | Healthy volunteers | Regular | Separate formulation | NR | NR | 0-infinity |
|  |  | R | 8 |  |  | Single dose |  |  |  |  |  |  |
|  |  | R | 8 |  |  | Steady state |  |  |  |  |  |  |
|  |  | R | 8 |  |  | Steady state |  |  |  |  |  |  |
|  |  | R | 8 | 450mg |  | Single dose |  |  |  |  |  |  |
|  |  | R | 8 |  |  | Single dose |  |  |  |  |  |  |
|  |  | R | 8 |  |  | Steady state |  |  |  |  |  |  |
|  |  | R | 8 |  |  | Steady state |  |  |  |  |  |  |
| Garg 1988^20^ | 4 | R | 11 | 450mg | NR | Single dose | TB patients | Regular | Separate formulation | NR | NR | 0-infinity |
|  |  | R | 10 |  |  |  |  |  |  |  |  |  |
|  |  | RH | 9 |  |  |  |  |  |  |  |  |  |
|  |  | RH | 10 |  |  |  |  |  |  |  |  |  |
| Gurumurthy 2004^21^ | 3 | RHZE | 13 | 450mg | B | Steady state | TB patients | Regular | Separate formulation | Negative | NR | 0-infinity |
|  |  | RHZE | 13 |  |  |  | Healthy volunteers |  |  | Positive |  |  |
|  |  | RHZE | 15 |  |  |  | HIV TB |  |  | Positive |  |  |
| Hao 2014^22^ | 8 | RH | 18 | 600mg | B | Single dose | Healthy volunteers | Regular | Separate formulation | NR | NR | 0-24 |
|  |  | RH | 18 |  |  |  |  |  |  |  |  |  |
|  |  | RH | 18 |  |  |  |  |  |  |  |  |  |
|  |  | RH | 18 |  |  |  |  |  | FDC |  |  |  |
|  |  | RH | 18 |  |  |  |  |  |  |  |  |  |
|  |  | RH | 18 |  |  |  |  |  |  |  |  |  |
|  |  | RH | 18 |  |  |  |  |  |  |  |  |  |
|  |  | RH | 18 |  |  |  |  |  |  |  |  |  |
| Hemanth Kumar 2016^23^ | 1 | RHZE S | 101 | < 60kg: 450mg; >= 60kg: 600mg | B | Steady state | TB patients | Intermittent | NR | Mixed population | Mixed population | 0-infinity |
| Israili 1986 ^24^ | 4 | RHE | 26 | 600mg | B | Single dose | TB patients | Regular | Separate formulation | NR | NR | 0-infinity |
|  |  | RHE | 26 |  |  | Steady state |  |  |  |  |  |  |
|  |  | RHE | 26 |  |  |  |  |  |  |  |  |  |
|  |  | RHE | 26 |  |  |  |  |  |  |  |  |  |
| Jaruratanasirikul 1996^25^ | 2 | R A | 11 | 300mg | A | Steady state | Healthy volunteers | Regular | Separate formulation | Positive | NR | 0-24 |
|  |  | R F | 11 |  |  |  |  |  |  |  |  |  |
| Jaruratanasirikul 2001^26^ | 2 | R | 11 | 600mg | B | Single dose | Healthy volunteers | Regular | Separate formulation | Positive | NR | 0-24 |
|  |  | R | 11 |  |  |  |  |  |  |  |  |  |
| Jian Xu 2013^27^ | 2 | RHZE | 18 | 600mg | B | Single dose | Healthy volunteers | Regular | FDC | NR | NR | 0-24 |
|  |  | RHZE | 18 |  |  |  |  |  | Separate formulation |  |  |  |
| Kelkar 1998^28^ | 1 | R | 6 | 150mg | A | Single dose | Healthy volunteers | Regular | NR | NR | NR | 0-infinity |
| Koegelenberg 2013^29^ | 1 | RHZE | 10 | < 37kg 300mg; 38-54kg 450mg; 55-70kg 600mg; >70kg 750mg | B | Steady state | TB patients | Regular | FDC | NR | NR | 0-24 |
| Loos 1985^30^ | 1 | RHE | 6 | 600mg | B | Steady state | TB patients | Regular | Separate formulation | NR | NR | 0-infinity |
| Lopez-Cortes 2002^31^ | 4 | RHZ ART | 8 | <50kg 480mg; 51-64kg 600mg; >= 65kg 720 | B | Steady state | TB patients | Regular | FDC | Positive | NR | 0-12 |
|  |  | RHZ ART | 8 |  |  |  |  |  |  |  |  |  |
|  |  | RHZ | 8 |  |  |  |  |  |  |  |  |  |
|  |  | RHZ ART | 8 |  |  |  |  |  |  |  |  |  |
|  |  | RHZE | 87 |  |  |  |  |  |  |  |  |  |
| McIlleron 2002^32^ | 2 | RHZE | 61 | 30-37kg 300mg; 38-54kg 450mg; 55-70kg 600mg; >70kg 750mg | B | Single dose | TB patients | Intermittent | FDC | Positive | NR | 0-12 |
|  |  | RHZE | 58 |  |  | Steady state |  |  |  |  |  |  |
| McIlleron 1999^33^ | 10 | RHZE | 19 | 600mg | B | Single dose | Healthy volunteers | Regular | FDC | NR | NR | 0-12 |
|  |  | RHZE | 19 |  |  |  |  |  | Separate formulation |  |  |  |
|  |  | RHZE | 24 |  |  |  |  |  | FDC |  |  | 0-24 |
|  |  | RHZE | 24 |  |  |  |  |  | Separate formulation |  |  |  |
|  |  | RHZE | 23 |  |  |  |  |  | FDC |  |  |  |
|  |  | RHZE | 23 |  |  |  |  |  | Separate formulation |  |  |  |
|  |  | RHZE | 24 |  |  |  |  |  | FDC |  |  |  |
|  |  | RHZE | 24 |  |  |  |  |  | Separate formulation |  |  |  |
|  |  | RHZE | 23 |  |  |  |  |  | FDC |  |  |  |
|  |  | RHZE | 23 |  |  |  |  |  | Separate formulation |  |  |  |
| McIlleron 2007^34^ | 2 | RHZ | 22 | 600mg | B | Single dose | Healthy volunteers | Regular | FDC | NR | NR | 0-infinity |
|  |  | RHZ GATI | 22 |  |  |  |  |  |  |  |  |  |
| McIlleron 2006^35^ | 2 | RHZE | 83 | 10.9mg/kg | B | Steady state | TB patients | Regular | NR | Mixed population | NR | 0-infinity |
|  |  | RHZE | 42 |  |  |  |  |  |  |  |  |  |
| Medellin-Garibay 2015^36^ | 3 | RHZ | 24 | 450mg < 50kg, 600mg >50 kg | B | Single dose | TB patients | Regular | FDC | Negative | NR | 0-24 |
|  |  | RHZ | 24 |  |  |  | TB patients |  |  |  | Diabetic | 0-24 |
|  |  | RHZ | 24 |  |  |  | Healthy volunteers |  |  |  | Non diabetic | 0-24 |
| Milan-Segovia 2010^37^ | 2 | RHZ | 18 | 600mg | B | Single dose | Healthy volunteers | Regular | FDC | NR | NR | 0-12 |
|  |  | RHZ | 18 |  |  |  |  |  |  |  |  |  |
| Nyazema 1999^38^ | 3 | RH | 10 | 450mg | A | Single dose | Healthy volunteers | Regular | FDC | NR | NR | 0-infinity |
|  |  | RH | 10 |  |  |  |  |  |  |  |  |  |
|  |  | RH | 10 |  |  |  |  |  | Separate formulation |  |  |  |
| Orisakwe 2001^39^ | 2 | R | 5 | 600mg | B | Single dose | Healthy volunteers | Regular | Separate formulation | NR | NR | 0-24 |
|  |  | R C | 5 |  |  |  |  |  |  |  |  |  |
| Orisakwe 1996^40^ | 2 | R | 6 | 600mg | B | Single dose | Healthy volunteers | Regular | Separate formulation | NR | NR | 0-24 |
|  |  | R | 6 |  |  |  |  |  |  |  |  | 0-infinity |
| Padgaonkar 1999^41^ | 3 | RH | 12 | 450mg | A | Single dose | Healthy volunteers | Regular | Separate formulation | NR | NR | 0-24 |
|  |  | RH | 12 |  |  |  |  |  |  |  |  |  |
|  |  | RH | 12 |  |  |  |  |  |  |  |  |  |
| Pahkla 1999^42^ | 3 | R | 19 | 600mg | B | Single dose | Healthy volunteers | Regular | Separate formulation | NR | Non diabetic | 0-24 |
|  |  | R | 19 |  |  |  |  |  |  |  |  |  |
|  |  | R | 19 |  |  |  |  |  |  |  |  |  |
| Panchagnula 2003^43^ | 2 | RHZE | 14 | 450mg | A | Single dose | Healthy volunteers | Regular | FDC | NR | NR | 0-24 |
|  |  | RHZE | 14 |  |  |  |  |  | Separate formulation |  |  |  |
| Pargal 2001^44^ | 2 | R | 12 | 450mg | NR | Single dose | Healthy volunteers | Regular | Separate formulation | NR | NR | 0-24 |
|  |  | R | 11 | 600mg |  |  |  |  |  |  |  |  |
| Peloquin 1999^45^ | 4 | RHZE | 14 | 600mg | B | Single dose | Healthy volunteers | Regular | Separate formulation | Negative | NR | 0-24 |
|  |  | RHZE | 14 |  |  |  |  |  |  |  |  |  |
|  |  | RHZE | 14 |  |  |  |  |  |  |  |  |  |
|  |  | RHZE | 14 |  |  |  |  |  |  |  |  |  |
| Peloquin 1997^46^ | 1 | RHZ | 24 | 600mg | B | Single dose | Healthy volunteers | Regular | Separate formulation | NR | NR | 0-infinity |
| Peloquin 2017 ^47^ | 3 | RHZE | 60 | 10mg/kg | B | Steady state | TB patients | Regular | FDC + additional rifampicin or placebo | Mixed population | Non diabetic | 0-24 |
|  |  |  | 60 | 15mg/kg | C |  |  |  |  |  | Non diabetic |  |
|  |  |  | 60 | 20mg/kg | C |  |  |  |  |  | Mixed population |  |
| Pillai 1999^48^ | 10 | RHZ | 19 | 600mg | NR | Single dose | Healthy volunteers | Regular | FDC | NR | NR | 0-12 |
|  |  | RHZE | 21 |  |  |  |  |  |  |  |  |  |
|  |  | RH | 22 |  |  |  |  |  |  |  |  |  |
|  |  | RH | 20 |  |  |  |  |  |  |  |  |  |
|  |  | RHZ | 21 |  |  |  |  |  |  |  |  |  |
|  |  | RH | 21 |  |  |  |  |  |  |  |  |  |
|  |  | RHZ | 22 | 450mg | NR | Single dose | Healthy volunteers | Regular | FDC | NR | NR | 0-12 |
|  |  | RHZE | 20 |  |  |  |  |  |  |  |  |  |
|  |  | RHZ | 22 |  |  |  |  |  |  |  |  |  |
|  |  | RHZ | 22 |  |  |  |  |  |  |  |  |  |
| Polk 2001^49^ | 2 | R | 11 | 600mg | B | Steady state | Healthy volunteers | Regular | Separate formulation | Negative | NR | 0-24 |
|  |  | R A | 11 |  |  |  |  |  |  |  |  |  |
| Potkar 1999^50^ | 1 | R | 8 | 300mg | A | Single dose | Healthy volunteers | Regular | Separate formulation | NR | NR | 0-infinity |
| Ribera 2001^51^ | 2 | R | 5 | 600mg | B | Steady state | TB patients | Regular | Separate formulation | Positive | NR | 0-24 |
|  |  | R N | 5 |  |  |  |  |  |  |  |  |  |
| Ribera 2007^52^ | 2 | RHZ | 22 | 600mg | B | Steady state | TB patients | Regular | FDC | Positive | NR | 0-24 |
|  |  | RH ART | 18 |  |  |  |  |  |  |  |  |  |
| Ruslami 2010^53^ | 2 | RHZE | 18 | 450mg | B | Steady state | TB patients | Regular | Separate formulation | Negative | Diabetic | 0-24 |
|  |  | RHZE | 18 |  |  |  |  |  |  |  | Non diabetic | 0-24 |
| Ruslami 2007^54^ | 2 | RHZE | 24 | 450mg | B | Steady state | TB patients | Regular | Separate formulation | Mixed population | NR | 0-24 |
|  |  | RHZE | 23 | 600mg | C |  |  |  |  |  |  |  |
| Saktiawati 2016^55^ | 2 | RHZE | 20 | 10mg/kg | B | Single dose | TB patients | Regular | Separate formulation | Mixed population | Mixed population | 0-24 |
|  |  | RHZE | 20 |  |  |  |  |  |  |  |  |  |
| Saleri 2012^56^ | 3 | RHZE | 16 | 10mg/kg | B | Steady state | TB patients | Regular | FDC | Positive | NR | 0-24 |
|  |  | RHZE ART | 16 |  |  |  |  |  |  |  |  |  |
|  |  | RHZE ART | 16 |  |  |  |  |  |  |  |  |  |
| Schall 1995^57^ | 2 | RHE | 20 | 600mg | NR | Single dose | Healthy volunteers | Regular | Separate formulation | NR | NR | 0-infinity |
|  |  | RHE | 20 |  |  |  |  |  | FDC |  |  |  |
| Shaheen 2012^58^ | 1 | RHZE | 20 | weight based ~ 10mg/kg | B | Steady state | TB patients | Regular | FDC | NR | NR | 0-12 |
| Sirgel 2005^59^ | 1 | R | 14 | 600mg | B | Single dose | TB patients | Regular | Separate formulation | NR | NR | 0-infinity |
| Sreenivasa 2001^60^ | 1 | R | 4 | 300mg | A | Single dose | Healthy volunteers | Regular | Separate formulation | NR | NR | 0-24 |
| Sturkenboom 2015 ^61^ | 1 | R + various | 33 | 8-12mg/kg | B | Steady state | TB patients | Regular | Separate formulation | Mixed population | Mixed population | 0-24 |
| Sturkenboom 2016 ^62^ | 1 | R + various | 66 | 8-12mg/kg | B | Steady state | TB patients | Regular | Separate formulation | Mixed population | Mixed population | 0-24 |
| te Brake 2015^63^ | 1 | RHZE | 36 | 10mg/kg | B | Steady state | TB patients | Regular | Separate formulation | Mixed population | NR | 0-24 |
| Tostmann 2013^64^ | 1 | RHZE | 20 | 450mg < 50kg, 600mg >50 kg | B | Steady state | TB patients | Regular | FDC | Mixed population | NR | 0-24 |
| van Crevel 2004^65^ | 4 | RHZE | 20 | 450mg | B | Single dose | Healthy volunteers | Regular | NR | NR | NR | 0-24 |
|  |  | RHZE | 20 |  |  |  |  |  |  |  |  | 0-24 |
|  |  | RHZE | 20 |  |  |  |  |  |  |  |  | 0-24 |
|  |  | RHZE | 20 |  |  |  |  |  |  |  |  | 0-24 |
| van Oosterhout 2015^66^ | 3 | RHZE | 41 | <38kg 300mg, <55kg 450mg, <75kg 600mg, >75kg 750mg | B | Steady state | TB patients | Regular | FDC | Mixed population | NR | 0-infinity |
| Weiner 2010^67^ | 2 | RZ M, E or H | 72 | < 45kg 450mg; > 45kg 600mg | B | Steady state | TB patients | Intermittent | Separate formulation | Mixed population | NR | 0-24 |
|  |  | RZ M, E or H | 16 | < 45kg 450mg; > 45kg 600mg |  |  | Healthy volunteers |  |  |  |  |  |
| Yunivita 2016^68^ | 2 | RHZE D | 11 | 750mg | C | Steady state | TB patients | Regular | Separate formulation | Mixed population | NR | 0-24 |
|  |  | RHZE D | 9 | 900mg |  |  |  |  |  |  |  |  |
| Zhu 2015^69^ | 7 | RHZE | 18 | 600mg | B | Single dose | Healthy volunteers | Regular | FDC | NR | Non diabetic | 0-24 |
|  |  | RHZE | 18 |  |  |  |  |  | Separate formulation |  |  |  |
|  |  | RH | 18 |  |  |  |  |  | FDC |  |  |  |
|  |  | RH | 18 |  |  |  |  |  | Separate formulation |  |  |  |
|  |  | RH | 20 |  |  |  |  |  | FDC |  |  |  |
|  |  | RH | 18 |  |  |  |  |  | Separate formulation |  |  |  |
|  |  | RH | 18 |  |  |  |  |  | FDC |  |  |  |
| Zwolska 2002^70^ | 2 | RHZ | 16 | 600mg | B | Single dose | Healthy volunteers | Regular | FDC | NR | NR | 0-24 |
|  |  | RHZ | 16 |  |  |  |  |  | Separate formulation |  |  |  |
| Total | **179** |  | **3477** |  |  |  |  |  |  |  |  |  |
| Author & Year | Study arms | Regimen | Number of subjects in study arm | Dose of rifampicin | Weight-based dosing category* | Schedule | Study population | Dosing frequency | Formulation | HIV Status | Diabetes status | AUC timeframe |
| Acocella 1988^1^ | 2 | RHZ | 10 | 600mg | B | Single dose | Healthy volunteers | Regular | Separate formulation | NR | NR | 0-24 |
|  |  | RHZ | 10 |  |  |  |  |  | FDC |  |  |  |
| Acocella 1993^2^ | 2 | RHZ | 6 | 600mg | NR | Single dose | Healthy volunteers | Regular | Separate formulation | NR | NR | 0-infinity |
|  |  | RHZ | 6 |  |  |  |  |  | FDC |  |  |  |
| Acocella2 1988^3^ | 2 | RHZ | 13 | Mean dose 11.5mg/kg | B | Steady state | TB patients | Regular | FDC | NR | NR | 0-12 |
|  |  | RHZ | 13 |  |  |  |  |  | FDC |  |  | 0-infinity |
| Agrawal 2002^4^ | 2 | RHZE | 14 | 450mg | B | Single dose | Healthy volunteers | Regular | FDC | NR | NR | 0-24 |
|  |  | RHZE | 14 |  |  |  |  |  | Separate formulation |  |  |  |
| Agrawal 2004^5^ | 2 | RHZE | 22 | 600mg | NR | Single dose | Healthy volunteers | Regular | FDC | NR | NR | 0-24 |
|  |  | RHZE | 22 |  |  |  |  |  | Separate formulation |  |  |  |
| Agrawal 2001^6^ | 2 | RHZE | 14 | 450mg | NR | Single dose | Healthy volunteers | Regular | FDC | NR | NR | 0-infinity |
|  |  | RHZE | 14 |  |  |  |  |  | Separate formulation |  |  |  |
| Agrawal2 2002^7^ | 2 | RHZE | 14 | 450mg | NR | Single dose | Healthy volunteers | Regular | FDC | NR | NR | 0-24 |
|  |  | RHZE | 14 |  |  |  |  |  | Separate formulation |  |  |  |
| Ahn 2003^8^ | 1 | RHZ | 9 | 450mg | A | Single dose | Healthy volunteers | Regular | Separate formulation | NR | NR | 0-infinity |
| Ashokraj 2005^9^ | 2 | RHE | 20 | 450mg | NR | Single dose | Healthy volunteers | Regular | FDC | Negative | NR | 0-24 |
|  |  | RHE | 20 |  |  |  |  |  |  |  |  |  |
| Avachat 2011^10^ | 1 | RH | 8 | 300mg | NR | Single dose | Healthy volunteers | Regular | Separate formulation | NR | NR | 0-infinity |
| Babalik 2013^11^ | 1 | RHZE | 21 | 600mg | B | Single dose | TB TB patients | Regular | Separate formulation | Negative | NR | 0-24 |
| Boeree 2015^12^ | 5 | RHZE | 8 | 10mg/kg | B | Steady state | TB patients | Regular | NR | Mixed population | NR | 0-24 |
|  |  | RHZE | 15 | 20mg/kg | C | Steady state | TB patients | Regular | NR | Mixed population | NR | 0-24 |
|  |  | RHZE | 15 | 25mg/kg |  |  |  |  |  |  |  |  |
|  |  | RHZE | 15 | 30mg/kg |  |  |  |  |  |  |  |  |
|  |  | RHZE | 15 | 35mg/kg |  |  |  |  |  |  |  |  |
| Boeree 2017 ^13^ | 5 | RHZE | 20 | 35mg/kg | C | Steady state | TB patients | Regular | NR | Mixed population | NR |  |
|  |  | RM | 19 | 20mg/kg | C |  |  |  |  |  |  |  |
|  |  | RS | 20 | 20mg/kg | C |  |  |  |  |  |  |  |
|  |  | RS | 20 | 10mg/kg | B |  |  |  |  |  |  |  |
|  |  | RHZE | 19 | 10mg/kg | B |  |  |  |  |  |  |  |
| Burhan 2013^14^ | 1 | RHZ | 9 | 30-39kg 300mg; 40-54kg 450mg; 55-70kg 600mg, >70kg 750mg | B | Steady state | TB patients | Regular | FDC | Mixed population | Mixed population | 0-24 |
| Chik 2010^15^ | 2 | R | 14 | 300mg | A | Single dose | Healthy volunteers | Regular | Separate formulation | Negative | NR | 0-24 |
|  |  | R | 14 |  |  |  |  |  |  |  |  |  |
| Choudhri 1997^16^ | 4 | RHZ | 29 | 600mg | B | Steady state | TB patients | Regular | Separate formulation | Positive | NR | 0-12 |
|  |  | RHZ | 29 |  |  |  |  |  |  | Negative |  |  |
|  |  | RHZ | 29 |  |  |  |  |  |  | NR |  |  |
|  |  | RHZ | 29 |  |  |  |  |  |  |  |  |  |
| Drusano 1986^17^ | 1 | R | 10 | 600mg | B | Steady state | Healthy volunteers | Regular | Separate formulation | NR | NR | 0-24 |
| Flores-Murrieta 1994^18^ | 1 | R | 8 | 600mg | B | Single dose | Healthy volunteers | Regular | Separate formulation | NR | NR | 0-24 |
| Garg 1991^19^ | 8 | R | 8 | 300mg | A | Single dose | Healthy volunteers | Regular | Separate formulation | NR | NR | 0-infinity |
|  |  | R | 8 |  |  | Single dose |  |  |  |  |  |  |
|  |  | R | 8 |  |  | Steady state |  |  |  |  |  |  |
|  |  | R | 8 |  |  | Steady state |  |  |  |  |  |  |
|  |  | R | 8 | 450mg |  | Single dose |  |  |  |  |  |  |
|  |  | R | 8 |  |  | Single dose |  |  |  |  |  |  |
|  |  | R | 8 |  |  | Steady state |  |  |  |  |  |  |
|  |  | R | 8 |  |  | Steady state |  |  |  |  |  |  |
| Garg 1988^20^ | 4 | R | 11 | 450mg | NR | Single dose | TB patients | Regular | Separate formulation | NR | NR | 0-infinity |
|  |  | R | 10 |  |  |  |  |  |  |  |  |  |
|  |  | RH | 9 |  |  |  |  |  |  |  |  |  |
|  |  | RH | 10 |  |  |  |  |  |  |  |  |  |
| Gurumurthy 2004^21^ | 3 | RHZE | 13 | 450mg | B | Steady state | TB patients | Regular | Separate formulation | Negative | NR | 0-infinity |
|  |  | RHZE | 13 |  |  |  | Healthy volunteers |  |  | Positive |  |  |
|  |  | RHZE | 15 |  |  |  | HIV TB |  |  | Positive |  |  |
| Hao 2014^22^ | 8 | RH | 18 | 600mg | B | Single dose | Healthy volunteers | Regular | Separate formulation | NR | NR | 0-24 |
|  |  | RH | 18 |  |  |  |  |  |  |  |  |  |
|  |  | RH | 18 |  |  |  |  |  |  |  |  |  |
|  |  | RH | 18 |  |  |  |  |  | FDC |  |  |  |
|  |  | RH | 18 |  |  |  |  |  |  |  |  |  |
|  |  | RH | 18 |  |  |  |  |  |  |  |  |  |
|  |  | RH | 18 |  |  |  |  |  |  |  |  |  |
|  |  | RH | 18 |  |  |  |  |  |  |  |  |  |
| Hemanth Kumar 2016^23^ | 1 | RHZE S | 101 | < 60kg: 450mg; >= 60kg: 600mg | B | Steady state | TB patients | Intermittent | NR | Mixed population | Mixed population | 0-infinity |
| Israili 1986 ^24^ | 4 | RHE | 26 | 600mg | B | Single dose | TB patients | Regular | Separate formulation | NR | NR | 0-infinity |
|  |  | RHE | 26 |  |  | Steady state |  |  |  |  |  |  |
|  |  | RHE | 26 |  |  |  |  |  |  |  |  |  |
|  |  | RHE | 26 |  |  |  |  |  |  |  |  |  |
| Jaruratanasirikul 1996^25^ | 2 | R A | 11 | 300mg | A | Steady state | Healthy volunteers | Regular | Separate formulation | Positive | NR | 0-24 |
|  |  | R F | 11 |  |  |  |  |  |  |  |  |  |
| Jaruratanasirikul 2001^26^ | 2 | R | 11 | 600mg | B | Single dose | Healthy volunteers | Regular | Separate formulation | Positive | NR | 0-24 |
|  |  | R | 11 |  |  |  |  |  |  |  |  |  |
| Jian Xu 2013^27^ | 2 | RHZE | 18 | 600mg | B | Single dose | Healthy volunteers | Regular | FDC | NR | NR | 0-24 |
|  |  | RHZE | 18 |  |  |  |  |  | Separate formulation |  |  |  |
| Kelkar 1998^28^ | 1 | R | 6 | 150mg | A | Single dose | Healthy volunteers | Regular | NR | NR | NR | 0-infinity |
| Koegelenberg 2013^29^ | 1 | RHZE | 10 | < 37kg 300mg; 38-54kg 450mg; 55-70kg 600mg; >70kg 750mg | B | Steady state | TB patients | Regular | FDC | NR | NR | 0-24 |
| Loos 1985^30^ | 1 | RHE | 6 | 600mg | B | Steady state | TB patients | Regular | Separate formulation | NR | NR | 0-infinity |
| Lopez-Cortes 2002^31^ | 4 | RHZ ART | 8 | <50kg 480mg; 51-64kg 600mg; >= 65kg 720 | B | Steady state | TB patients | Regular | FDC | Positive | NR | 0-12 |
|  |  | RHZ ART | 8 |  |  |  |  |  |  |  |  |  |
|  |  | RHZ | 8 |  |  |  |  |  |  |  |  |  |
|  |  | RHZ ART | 8 |  |  |  |  |  |  |  |  |  |
|  |  | RHZE | 87 |  |  |  |  |  |  |  |  |  |
| McIlleron 2002^32^ | 2 | RHZE | 61 | 30-37kg 300mg; 38-54kg 450mg; 55-70kg 600mg; >70kg 750mg | B | Single dose | TB patients | Intermittent | FDC | Positive | NR | 0-12 |
|  |  | RHZE | 58 |  |  | Steady state |  |  |  |  |  |  |
| McIlleron 1999^33^ | 10 | RHZE | 19 | 600mg | B | Single dose | Healthy volunteers | Regular | FDC | NR | NR | 0-12 |
|  |  | RHZE | 19 |  |  |  |  |  | Separate formulation |  |  |  |
|  |  | RHZE | 24 |  |  |  |  |  | FDC |  |  | 0-24 |
|  |  | RHZE | 24 |  |  |  |  |  | Separate formulation |  |  |  |
|  |  | RHZE | 23 |  |  |  |  |  | FDC |  |  |  |
|  |  | RHZE | 23 |  |  |  |  |  | Separate formulation |  |  |  |
|  |  | RHZE | 24 |  |  |  |  |  | FDC |  |  |  |
|  |  | RHZE | 24 |  |  |  |  |  | Separate formulation |  |  |  |
|  |  | RHZE | 23 |  |  |  |  |  | FDC |  |  |  |
|  |  | RHZE | 23 |  |  |  |  |  | Separate formulation |  |  |  |
| McIlleron 2007^34^ | 2 | RHZ | 22 | 600mg | B | Single dose | Healthy volunteers | Regular | FDC | NR | NR | 0-infinity |
|  |  | RHZ GATI | 22 |  |  |  |  |  |  |  |  |  |
| McIlleron 2006^35^ | 2 | RHZE | 83 | 10.9mg/kg | B | Steady state | TB patients | Regular | NR | Mixed population | NR | 0-infinity |
|  |  | RHZE | 42 |  |  |  |  |  |  |  |  |  |
| Medellin-Garibay 2015^36^ | 3 | RHZ | 24 | 450mg < 50kg, 600mg >50 kg | B | Single dose | TB patients | Regular | FDC | Negative | NR | 0-24 |
|  |  | RHZ | 24 |  |  |  | TB patients |  |  |  | Diabetic | 0-24 |
|  |  | RHZ | 24 |  |  |  | Healthy volunteers |  |  |  | Non diabetic | 0-24 |
| Milan-Segovia 2010^37^ | 2 | RHZ | 18 | 600mg | B | Single dose | Healthy volunteers | Regular | FDC | NR | NR | 0-12 |
|  |  | RHZ | 18 |  |  |  |  |  |  |  |  |  |
| Nyazema 1999^38^ | 3 | RH | 10 | 450mg | A | Single dose | Healthy volunteers | Regular | FDC | NR | NR | 0-infinity |
|  |  | RH | 10 |  |  |  |  |  |  |  |  |  |
|  |  | RH | 10 |  |  |  |  |  | Separate formulation |  |  |  |
| Orisakwe 2001^39^ | 2 | R | 5 | 600mg | B | Single dose | Healthy volunteers | Regular | Separate formulation | NR | NR | 0-24 |
|  |  | R C | 5 |  |  |  |  |  |  |  |  |  |
| Orisakwe 1996^40^ | 2 | R | 6 | 600mg | B | Single dose | Healthy volunteers | Regular | Separate formulation | NR | NR | 0-24 |
|  |  | R | 6 |  |  |  |  |  |  |  |  | 0-infinity |
| Padgaonkar 1999^41^ | 3 | RH | 12 | 450mg | A | Single dose | Healthy volunteers | Regular | Separate formulation | NR | NR | 0-24 |
|  |  | RH | 12 |  |  |  |  |  |  |  |  |  |
|  |  | RH | 12 |  |  |  |  |  |  |  |  |  |
| Pahkla 1999^42^ | 3 | R | 19 | 600mg | B | Single dose | Healthy volunteers | Regular | Separate formulation | NR | Non diabetic | 0-24 |
|  |  | R | 19 |  |  |  |  |  |  |  |  |  |
|  |  | R | 19 |  |  |  |  |  |  |  |  |  |
| Panchagnula 2003^43^ | 2 | RHZE | 14 | 450mg | A | Single dose | Healthy volunteers | Regular | FDC | NR | NR | 0-24 |
|  |  | RHZE | 14 |  |  |  |  |  | Separate formulation |  |  |  |
| Pargal 2001^44^ | 2 | R | 12 | 450mg | NR | Single dose | Healthy volunteers | Regular | Separate formulation | NR | NR | 0-24 |
|  |  | R | 11 | 600mg |  |  |  |  |  |  |  |  |
| Peloquin 1999^45^ | 4 | RHZE | 14 | 600mg | B | Single dose | Healthy volunteers | Regular | Separate formulation | Negative | NR | 0-24 |
|  |  | RHZE | 14 |  |  |  |  |  |  |  |  |  |
|  |  | RHZE | 14 |  |  |  |  |  |  |  |  |  |
|  |  | RHZE | 14 |  |  |  |  |  |  |  |  |  |
| Peloquin 1997^46^ | 1 | RHZ | 24 | 600mg | B | Single dose | Healthy volunteers | Regular | Separate formulation | NR | NR | 0-infinity |
| Peloquin 2017 ^47^ | 3 | RHZE | 60 | 10mg/kg | B | Steady state | TB patients | Regular | FDC + additional rifampicin or placebo | Mixed population | Non diabetic | 0-24 |
|  |  |  | 60 | 15mg/kg | C |  |  |  |  |  | Non diabetic |  |
|  |  |  | 60 | 20mg/kg | C |  |  |  |  |  | Mixed population |  |
| Pillai 1999^48^ | 10 | RHZ | 19 | 600mg | NR | Single dose | Healthy volunteers | Regular | FDC | NR | NR | 0-12 |
|  |  | RHZE | 21 |  |  |  |  |  |  |  |  |  |
|  |  | RH | 22 |  |  |  |  |  |  |  |  |  |
|  |  | RH | 20 |  |  |  |  |  |  |  |  |  |
|  |  | RHZ | 21 |  |  |  |  |  |  |  |  |  |
|  |  | RH | 21 |  |  |  |  |  |  |  |  |  |
|  |  | RHZ | 22 | 450mg | NR | Single dose | Healthy volunteers | Regular | FDC | NR | NR | 0-12 |
|  |  | RHZE | 20 |  |  |  |  |  |  |  |  |  |
|  |  | RHZ | 22 |  |  |  |  |  |  |  |  |  |
|  |  | RHZ | 22 |  |  |  |  |  |  |  |  |  |
| Polk 2001^49^ | 2 | R | 11 | 600mg | B | Steady state | Healthy volunteers | Regular | Separate formulation | Negative | NR | 0-24 |
|  |  | R A | 11 |  |  |  |  |  |  |  |  |  |
| Potkar 1999^50^ | 1 | R | 8 | 300mg | A | Single dose | Healthy volunteers | Regular | Separate formulation | NR | NR | 0-infinity |
| Ribera 2001^51^ | 2 | R | 5 | 600mg | B | Steady state | TB patients | Regular | Separate formulation | Positive | NR | 0-24 |
|  |  | R N | 5 |  |  |  |  |  |  |  |  |  |
| Ribera 2007^52^ | 2 | RHZ | 22 | 600mg | B | Steady state | TB patients | Regular | FDC | Positive | NR | 0-24 |
|  |  | RH ART | 18 |  |  |  |  |  |  |  |  |  |
| Ruslami 2010^53^ | 2 | RHZE | 18 | 450mg | B | Steady state | TB patients | Regular | Separate formulation | Negative | Diabetic | 0-24 |
|  |  | RHZE | 18 |  |  |  |  |  |  |  | Non diabetic | 0-24 |
| Ruslami 2007^54^ | 2 | RHZE | 24 | 450mg | B | Steady state | TB patients | Regular | Separate formulation | Mixed population | NR | 0-24 |
|  |  | RHZE | 23 | 600mg | C |  |  |  |  |  |  |  |
| Saktiawati 2016^55^ | 2 | RHZE | 20 | 10mg/kg | B | Single dose | TB patients | Regular | Separate formulation | Mixed population | Mixed population | 0-24 |
|  |  | RHZE | 20 |  |  |  |  |  |  |  |  |  |
| Saleri 2012^56^ | 3 | RHZE | 16 | 10mg/kg | B | Steady state | TB patients | Regular | FDC | Positive | NR | 0-24 |
|  |  | RHZE ART | 16 |  |  |  |  |  |  |  |  |  |
|  |  | RHZE ART | 16 |  |  |  |  |  |  |  |  |  |
| Schall 1995^57^ | 2 | RHE | 20 | 600mg | NR | Single dose | Healthy volunteers | Regular | Separate formulation | NR | NR | 0-infinity |
|  |  | RHE | 20 |  |  |  |  |  | FDC |  |  |  |
| Shaheen 2012^58^ | 1 | RHZE | 20 | weight based ~ 10mg/kg | B | Steady state | TB patients | Regular | FDC | NR | NR | 0-12 |
| Sirgel 2005^59^ | 1 | R | 14 | 600mg | B | Single dose | TB patients | Regular | Separate formulation | NR | NR | 0-infinity |
| Sreenivasa 2001^60^ | 1 | R | 4 | 300mg | A | Single dose | Healthy volunteers | Regular | Separate formulation | NR | NR | 0-24 |
| Sturkenboom 2015 ^61^ | 1 | R + various | 33 | 8-12mg/kg | B | Steady state | TB patients | Regular | Separate formulation | Mixed population | Mixed population | 0-24 |
| Sturkenboom 2016 ^62^ | 1 | R + various | 66 | 8-12mg/kg | B | Steady state | TB patients | Regular | Separate formulation | Mixed population | Mixed population | 0-24 |
| te Brake 2015^63^ | 1 | RHZE | 36 | 10mg/kg | B | Steady state | TB patients | Regular | Separate formulation | Mixed population | NR | 0-24 |
| Tostmann 2013^64^ | 1 | RHZE | 20 | 450mg < 50kg, 600mg >50 kg | B | Steady state | TB patients | Regular | FDC | Mixed population | NR | 0-24 |
| van Crevel 2004^65^ | 4 | RHZE | 20 | 450mg | B | Single dose | Healthy volunteers | Regular | NR | NR | NR | 0-24 |
|  |  | RHZE | 20 |  |  |  |  |  |  |  |  | 0-24 |
|  |  | RHZE | 20 |  |  |  |  |  |  |  |  | 0-24 |
|  |  | RHZE | 20 |  |  |  |  |  |  |  |  | 0-24 |
| van Oosterhout 2015^66^ | 3 | RHZE | 41 | <38kg 300mg, <55kg 450mg, <75kg 600mg, >75kg 750mg | B | Steady state | TB patients | Regular | FDC | Mixed population | NR | 0-infinity |
| Weiner 2010^67^ | 2 | RZ M, E or H | 72 | < 45kg 450mg; > 45kg 600mg | B | Steady state | TB patients | Intermittent | Separate formulation | Mixed population | NR | 0-24 |
|  |  | RZ M, E or H | 16 | < 45kg 450mg; > 45kg 600mg |  |  | Healthy volunteers |  |  |  |  |  |
| Yunivita 2016^68^ | 2 | RHZE D | 11 | 750mg | C | Steady state | TB patients | Regular | Separate formulation | Mixed population | NR | 0-24 |
|  |  | RHZE D | 9 | 900mg |  |  |  |  |  |  |  |  |
| Zhu 2015^69^ | 7 | RHZE | 18 | 600mg | B | Single dose | Healthy volunteers | Regular | FDC | NR | Non diabetic | 0-24 |
|  |  | RHZE | 18 |  |  |  |  |  | Separate formulation |  |  |  |
|  |  | RH | 18 |  |  |  |  |  | FDC |  |  |  |
|  |  | RH | 18 |  |  |  |  |  | Separate formulation |  |  |  |
|  |  | RH | 20 |  |  |  |  |  | FDC |  |  |  |
|  |  | RH | 18 |  |  |  |  |  | Separate formulation |  |  |  |
|  |  | RH | 18 |  |  |  |  |  | FDC |  |  |  |
| Zwolska 2002^70^ | 2 | RHZ | 16 | 600mg | B | Single dose | Healthy volunteers | Regular | FDC | NR | NR | 0-24 |
|  |  | RHZ | 16 |  |  |  |  |  | Separate formulation |  |  |  |
| Total | **179** |  | **3477** |  |  |  |  |  |  |  |  |  |

R: Rifampicin; H: Isoniazid; Z: Pyrazinamide; E: Ethambutol; C: Ciprofloxacin; S: SQ109; GATI: Gatifloxacin; M: Moxifloxacin; D: Dexamethasone; ART: Antiretroviral Therapy; N: Nevirapine; A: Amprenavir; NR: Not reported. FDC: Fixed-dose combination.

* Weight-based dosing category A: <8mg/kg; B: 8-12mg/kg; C:>12mg/kg.

**Table S2.** Meta-regression of variables influencing estimated rifampicin Cmax

| **Variable and category** | **Adjusted Cmax estimate (μg/ml)** | **95% confidence interval** | **SEM** | **p-value** |
| --- | --- | --- | --- | --- |
| Duration of therapy |  | | | |
| Single dose | 8.98 | 6.36 – 11.61 | 1.34 | NA |
| Steady state dosing (> 1 week) | 5.79 | 4.01 - 7.56 | 0.90 | 0.0004 |
| HIV status |  | | | |
| HIV negative | 9.25 | 7.48 – 11.02 | 0.90 | 0.77 |
| HIV positive | 10.62 | 8.86 – 12.39 | 0.90 | 0.07 |
| Mixed HIV population | 9.33 | 7.29 – 11.38 | 1.04 | 0.74 |
| TB status |  | | | |
| TB patients | 8.98 | 6.36 – 11.61 | 1.34 | 0.002 |
| Healthy volunteers | 6.39 | 4.72 – 8.07 | 0.85 | 0.002 |
| Drug combination |  | | | |
| Rifampicin monotherapy | 8.68 | 5.18 – 12.17 | 1.78 | 0.20 |
| RHZE | 6.71 | 3.24 – 10.20 | 1.77 | 0.86 |
| Diabetes status |  | | | |
| No diabetes | 12.54 | 9.17 – 15.93 | 1.73 | 0.27 |
| Diabetes | 14.78 | 10.20 – 19.36 | 2.33 | 0.99 |
| Dosing frequency |  | | | |
| Daily dosing | 7.99 | 6.35 – 9.63 | 0.84 | 0.24 |
| Intermittent dosing | 8.29 | 4.24 – 12.34 | 2.07 | 0.74 |

Meta-regression of all available variables found that treatment duration had a substantial and significant impact on estimated rifampicin Cmax.

Steady-state refers to dosing for ≥7 days to allow for saturation of first-pass metabolism and the establishment of metabolic autoinduction.

Cmax: maximum concentration of rifampicin in plasma. SEM: Standard error of the mean; P-values indicate significance of difference between pooled Cmax estimates and overall population estimate.

**Figure S1.** Preferred Reporting Items for Systematic Reviews and Meta-Analyses (PRISMA) selection flow diagram for studies of rifampicin pharmacokinetics to May 2017.


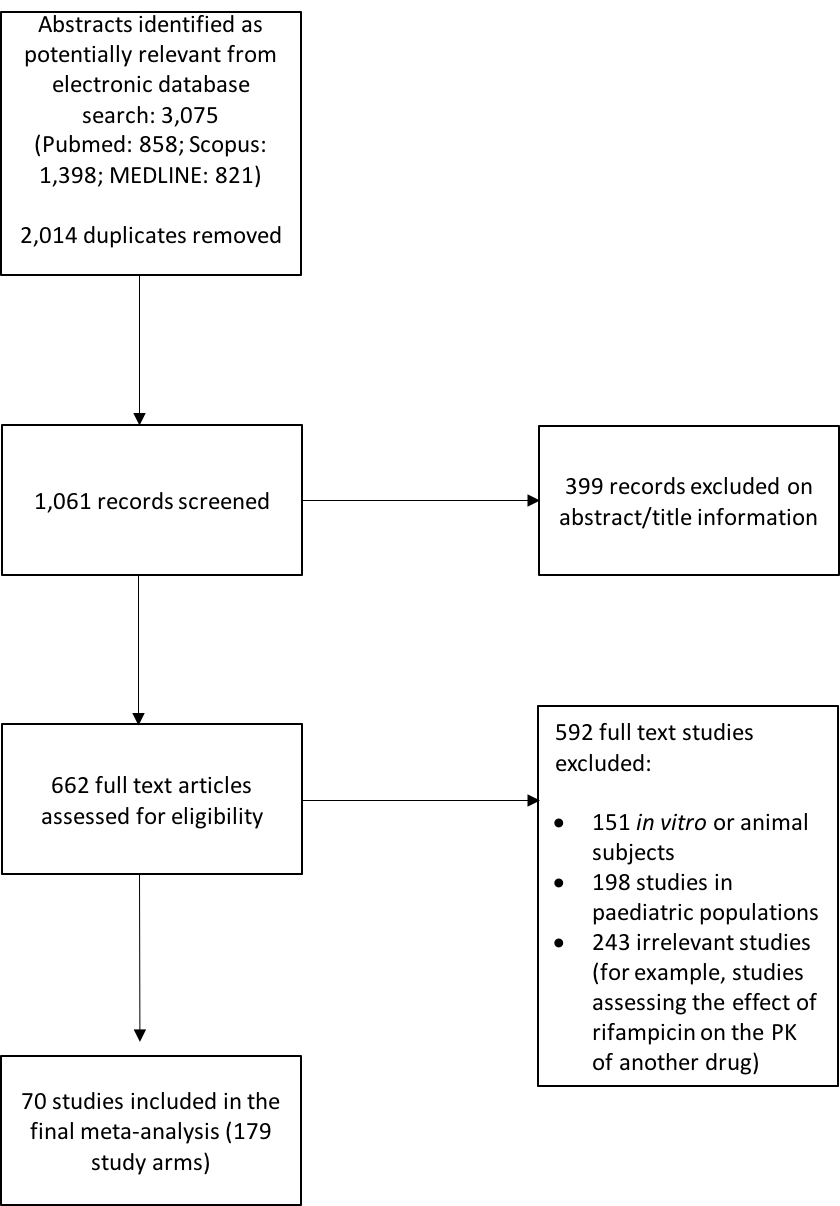


**Figure S2.** Forest plot displaying estimated rifampicin AUC after univariate analysis according to dosing duration.


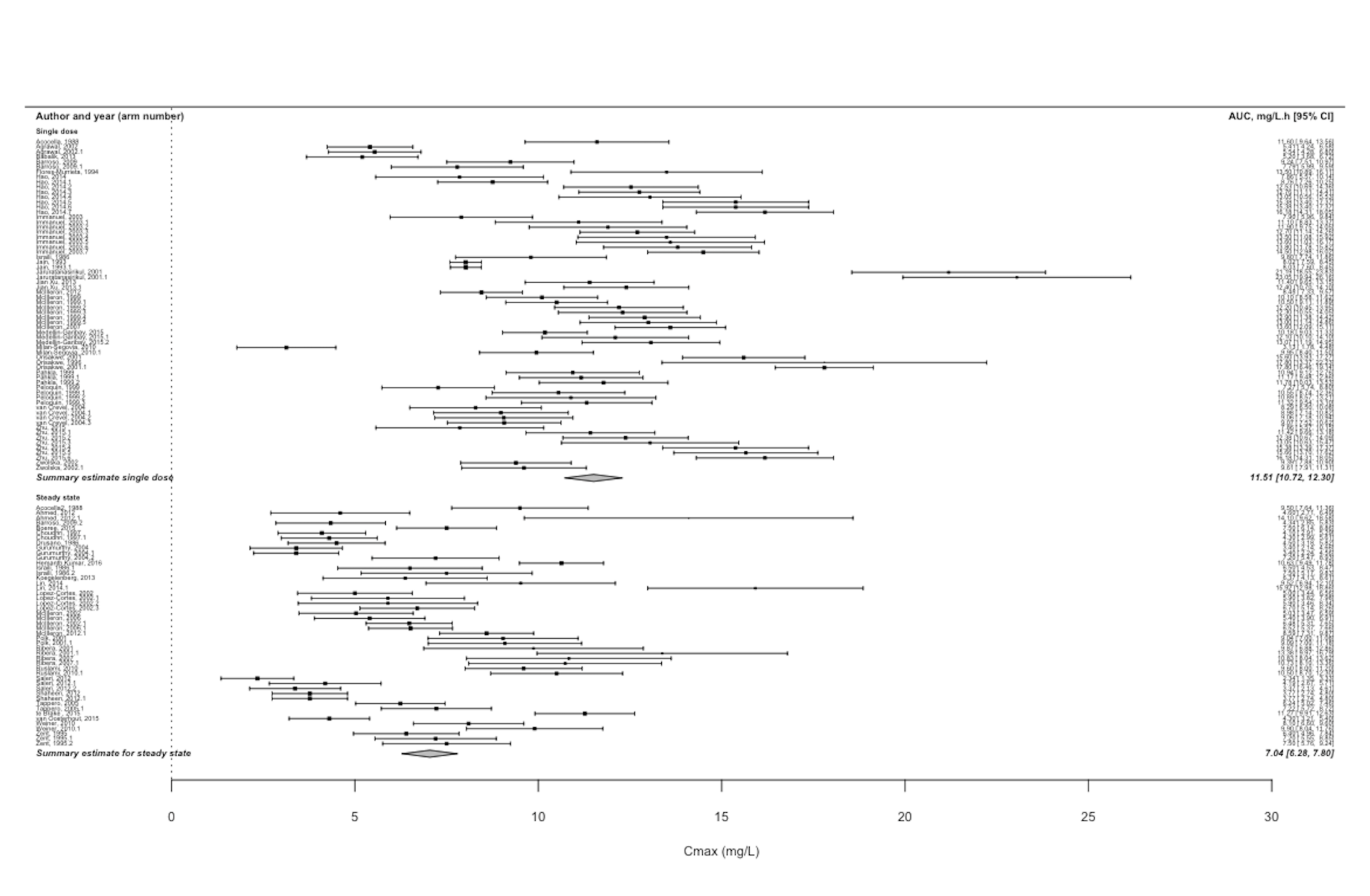


**References**

1. Acocella G, Nonis A, Gialdroni-Grassi G et al. Comparative bioavailability of isoniazid, rifampin, and pyrazinamide administered in free combination and in a fixed triple formulation designed for daily use in antituberculosis chemotherapy. I. Single-dose study. *Am Rev Respir Dis* 1988; **138**: 882-5.

2. Acocella G, Luisetti M, Grassi GG et al. Bioavailability of isoniazid, rifampicin and pyrazinamide (in free combination or fixed-triple formulation) in intermittent antituberculous chemotherapy. *Monaldi Arch Chest Dis* 1993; **48**: 205-9.

3. Acocella G, Nonis A, Perna G et al. Comparative bioavailability of isoniazid, rifampin, and pyrazinamide administered in free combination and in a fixed triple formulation designed for daily use in antituberculosis chemotherapy. II. Two-month, daily administration study. *Am Rev Respir Dis* 1988; **138**: 886-90.

4. Agrawal S, Kaur KJ, Singh I et al. Assessment of bioequivalence of rifampicin, isoniazid and pyrazinamide in a four drug fixed dose combination with separate formulations at the same dose levels. *Int J Pharm* 2002; **233**: 169-77.

5. Agrawal S, Singh I, Kaur KJ et al. Comparative bioavailability of rifampicin, isoniazid and pyrazinamide from a four drug fixed dose combination with separate formulations at the same dose levels. *Int J Pharm* 2004; **276**: 41-9.

6. Agrawal S, Kaul CL, Panchagnula R. Bioequivalence of isoniazid in a two drug fixed dose combination and in a single drug dosage form. *Pharmazie* 2001; **56**: 636-9.

7. Agrawal S, Singh I, Kaur KJ et al. Bioequivalence assessment of rifampicin, isoniazid and pyrazinamide in a fixed dose combination of rifampicin, isoniazid, pyrazinamide and ethambutol vs. separate formulations. *Int J Clin Pharmacol Ther* 2002; **40**: 474-81.

8. Ahn C, Oh KH, Kim K et al. Effect of peritoneal dialysis on plasma and peritoneal fluid concentrations of isoniazid, pyrazinamide, and rifampin. *Perit Dial Int* 2003; **23**: 362-7.

9. Ashokraj Y, Singh I, Kaur KJ et al. Establishment of a reference formulation for bioequivalence assessment of rifampicin-containing FDCs: an essential step towards improving tuberculosis treatment. *Int J Tuberc Lung Dis* 2005; **9**: 791-6.

10. Avachat AM, Bhise SB. Tailored release drug delivery system for rifampicin and isoniazid for enhanced bioavailability of rifampicin. *Pharm Dev Technol* 2011; **16**: 127-36.

11. Babalık A, Ulus IH, Bakirci N et al. Pharmacokinetics and serum concentrations of antimycobacterial drugs in adult Turkish patients. *Int J Tuberc Lung Dis* 2013; **17**: 1442-7.

12. Boeree MJ, Diacon AH, Dawson R et al. A dose-ranging trial to optimize the dose of rifampin in the treatment of tuberculosis. *Am J Respir Crit Care Med* 2015; **191**: 1058-65.

13. Boeree MJ, Heinrich N, Aarnoutse R et al. High-dose rifampicin, moxifloxacin, and SQ109 for treating tuberculosis: a multi-arm, multi-stage randomised controlled trial. *Lancet Infect Dis* 2017; **17**: 39-49.

14. Burhan E, Ruesen C, Ruslami R et al. Isoniazid, rifampin, and pyrazinamide plasma concentrations in relation to treatment response in Indonesian pulmonary tuberculosis patients. *Antimicrob Agents Chemother* 2013; **57**: 3614-9.

15. Chik Z, Basu RC, Pendek R et al. A bioequivalence comparison of two formulations of rifampicin (300- vs 150-mg capsules): An open-label, randomized, two-treatment, two-way crossover study in healthy volunteers. *Clin Ther* 2010; **32**: 1822-31.

16. Choudhri SH, Hawken M, Gathua S et al. Pharmacokinetics of antimycobacterial drugs in patients with tuberculosis, AIDS, and diarrhea. *Clin Infect Dis* 1997; **25**: 104-11.

17. Drusano GL, Townsend RJ, Walsh TJ et al. Steady-state serum pharmacokinetics of novobiocin and rifampin alone and in combination. *Antimicrob Agents Chemother* 1986; **30**: 42-5.

18. Flores-Murrieta FJ, Castañeda-Hernández G, Herrera JE et al. Determination of oral rifampin pharmacokinetic parameters in Mexicans and comparison with other populations: absence of evidence for interethnic variability. *Arch Med Res* 1994; **25**: 381-5.

19. Garg SK, Chakrabarti A, Panigrahi D et al. Comparative bioavailability and in-vitro antimicrobial activity of two different brands of rifampicin. *Eur J Drug Metab Pharmacokinet* 1991; **16**: 223-9.

20. Garg SK, Dhand R, Malik SK et al. Single dose kinetics of rifampicin and isoniazid in well-nourished and malnourished patients of tuberculosis. *Int J Clin Pharmacol Ther Toxicol* 1988; **26**: 417-20.

21. Gurumurthy P, Ramachandran G, Hemanth Kumar AK et al. Decreased bioavailability of rifampin and other antituberculosis drugs in patients with advanced human immunodeficiency virus disease. *Antimicrob Agents Chemother* 2004; **48**: 4473-5.

22. Hao LH, Guo SC, Liu CC et al. Comparative bioavailability of rifampicin and isoniazid in fixed-dose combinations and single-drug formulations. *Int J Tuberc Lung Dis* 2014; **18**: 1505-12.

23. Hemanth Kumar AK, Kannan T, Chandrasekaran V et al. Pharmacokinetics of thrice-weekly rifampicin, isoniazid and pyrazinamide in adult tuberculosis patients in India. *Int J Tuberc Lung Dis* 2016; **20**: 1236-41.

24. Israili ZH, Rogers CM, el-Attar H. Pharmacokinetics of antituberculosis drugs in patients. *J Clin Pharmacol* 1987; **27**: 78-83.

25. Jaruratanasirikul S, Kleepkaew A. Lack of effect of fluconazole on the pharmacokinetics of rifampicin in AIDS patients. *J Antimicrob Chemother* 1996; **38**: 877-80.

26. Jaruratanasirikul S, Sriwiriyajan S. Effect of indinavir on the pharmacokinetics of rifampicin in HIV-infected patients. *J Pharm Pharmacol* 2001; **53**: 409-12.

27. Xu J, Jin H, Zhu H et al. Oral bioavailability of rifampicin, isoniazid, ethambutol, and pyrazinamide in a 4-drug fixed-dose combination compared with the separate formulations in healthy Chinese male volunteers. *Clin Ther* 2013; **35**: 161-8.

28. Kelkar MS, Saraf AP, Bakhle DS et al. Pharmacokinetic profile of a new 3-azinomethyl rifamycin (SPA-S-565) in volunteers as compared with conventional rifampicin. *Int J Clin Pharmacol Res* 1998; **18**: 137-43.

29. Koegelenberg CF, Nortje A, Lalla U et al. The pharmacokinetics of enteral antituberculosis drugs in patients requiring intensive care. *S Afr Med J* 2013; **103**: 394-8.

30. Loos U, Musch E, Jensen JC et al. Pharmacokinetics of oral and intravenous rifampicin during chronic administration. *Klin Wochenschr* 1985; **63**: 1205-11.

31. Lopez-Cortes LF, Ruiz-Valderas R, Viciana P et al. Pharmacokinetic interactions between efavirenz and rifampicin in HIV-infected patients with tuberculosis. *Clin Pharmacokinet* 2002; **41**: 681-90.

32. McIlleron H, Wash P, Burger A et al. Widespread distribution of a single drug rifampicin formulation of inferior bioavailability in South Africa. *Int J Tuberc Lung Dis* 2002; **6**: 356-61.

33. McIlleron H, Gabriels G, Smith PJ et al. The development of a standardised screening protocol for the in vivo assessment of rifampicin bioavailability. *Int J Tuberc Lung Dis* 1999; **3**: S329-35; discussion S51-2.

34. McIlleron H, Norman J, Kanyok TP et al. Elevated gatifloxacin and reduced rifampicin concentrations in a single-dose interaction study amongst healthy volunteers. *J Antimicrob Chemother* 2007; **60**: 1398-401.

35. McIlleron H, Wash P, Burger A et al. Determinants of rifampin, isoniazid, pyrazinamide, and ethambutol pharmacokinetics in a cohort of tuberculosis patients. *Antimicrob Agents Chemother* 2006; **50**: 1170-7.

36. Medellín-Garibay SE, Cortez-Espinosa N, Milán-Segovia RC et al. Clinical Pharmacokinetics of Rifampin in Patients with Tuberculosis and Type 2 Diabetes Mellitus: Association with Biochemical and Immunological Parameters. *Antimicrob Agents Chemother* 2015; **59**: 7707-14.

37. Milán-Segovia RC, Domínguez-Ramírez AM, Jung-Cook H et al. Relative bioavailability of rifampicin in a three-drug fixed-dose combination formulation. *Int J Tuberc Lung Dis* 2010; **14**: 1454-60.

38. Nyazema NZ, Rabvukwa P, Gumbo J et al. Bioavailability of rifampicin in a separate formulation and fixed dose combination with isoniazid NIH: a case for a fixed dose combination (FDC) for the treatment of tuberculosis. *Cent Afr J Med* 1999; **45**: 141-4.

39. Orisakwe OE, Agbasi PU, Afonne OJ et al. Rifampicin pharmacokinetics with and without ciprofloxacin. *Am J Ther* 2001; **8**: 151-3.

40. Orisakwe OE, Ofoefule SI. Plasma and saliva concentrations of rifampicin in man after oral administration. *Tokai J Exp Clin Med* 1996; **21**: 45-9.

41. Padgaonkar KA, Revankar SN, Bhatt AD et al. Comparative bioequivalence study of rifampicin and isoniazid combinations in healthy volunteers. *Int J Tuberc Lung Dis* 1999; **3**: 627-31.

42. Pähkla R, Lambert J, Ansko P et al. Comparative bioavailability of three different preparations of rifampicin. *J Clin Pharm Ther* 1999; **24**: 219-25.

43. Panchagnula R, Sharma A, Agrawal S. Plasma pooling methodology as a faster and cheaper tool to evaluate bioequivalence of rifampicin component of FDCs of antitubercular drugs. *Pharmacol Res* 2003; **48**: 655-63.

44. Pargal A, Rani S. Non-linear pharmacokinetics of rifampicin in healthy Asian Indian volunteers. *Int J Tuberc Lung Dis* 2001; **5**: 70-9.

45. Peloquin CA, Namdar R, Singleton MD et al. Pharmacokinetics of rifampin under fasting conditions, with food, and with antacids. *Chest* 1999; **115**: 12-8.

46. Peloquin CA, Jaresko GS, Yong CL et al. Population pharmacokinetic modeling of isoniazid, rifampin, and pyrazinamide. *Antimicrob Agents Chemother* 1997; **41**: 2670-9.

47. Peloquin CA, Velasquez GE, Lecca L et al. Pharmacokinetic evidence to support increased doses of rifampin for tuberculosis: results from the HIRIF trial. *Antimicrob Agents Chemother* 2017.

48. Pillai G, Fourie PB, Padayatchi N et al. Recent bioequivalence studies on fixed-dose combination anti-tuberculosis drug formulations available on the global market. *Int J Tuberc Lung Dis* 1999; **3**: S309-16; discussion S17-21.

49. Polk RE, Brophy DF, Israel DS et al. Pharmacokinetic Interaction between amprenavir and rifabutin or rifampin in healthy males. *Antimicrob Agents Chemother* 2001; **45**: 502-8.

50. Potkar C, Gogtay N, Gokhale P et al. Phase I pharmacokinetic study of a new 3-azinomethyl-rifamycin (rifametane) as compared to rifampicin. *Chemotherapy* 1999; **45**: 147-53.

51. Ribera E, Pou L, Lopez RM et al. Pharmacokinetic interaction between nevirapine and rifampicin in HIV-infected patients with tuberculosis. *J Acquir Immune Defic Syndr* 2001; **28**: 450-3.

52. Ribera E, Azuaje C, Lopez RM et al. Pharmacokinetic interaction between rifampicin and the once-daily combination of saquinavir and low-dose ritonavir in HIV-infected patients with tuberculosis. *J Antimicrob Chemother* 2007; **59**: 690-7.

53. Ruslami R, Nijland HM, Adhiarta IG et al. Pharmacokinetics of antituberculosis drugs in pulmonary tuberculosis patients with type 2 diabetes. *Antimicrob Agents Chemother* 2010; **54**: 1068-74.

54. Ruslami R, Nijland HM, Alisjahbana B et al. Pharmacokinetics and tolerability of a higher rifampin dose versus the standard dose in pulmonary tuberculosis patients. *Antimicrob Agents Chemother* 2007; **51**: 2546-51.

55. Saktiawati AM, Sturkenboom MG, Stienstra Y et al. Impact of food on the pharmacokinetics of first-line anti-TB drugs in treatment-naive TB patients: a randomized cross-over trial. *J Antimicrob Chemother* 2016; **71**: 703-10.

56. Saleri N, Dembélé SM, Villani P et al. Systemic exposure to rifampicin in patients with tuberculosis and advanced HIV disease during highly active antiretroviral therapy in Burkina Faso. *J Antimicrob Chemother* 2012; **67**: 469-72.

57. Schall R, Müller FO, Duursema L et al. Relative bioavailability of rifampicin, isoniazid and ethambutol from a combination tablet vs. concomitant administration of a capsule containing rifampicin and a tablet containing isoniazid and ethambutol. *Arzneimittelforschung* 1995; **45**: 1236-9.

58. Shaheen A, Najmi MH, Saeed W et al. Pharmacokinetics of standard dose regimens of rifampicin in patients with pulmonary tuberculosis in Pakistan. *Scand J Infect Dis* 2012; **44**: 459-64.

59. Sirgel FA, Fourie PB, Donald PR et al. The early bactericidal activities of rifampin and rifapentine in pulmonary tuberculosis. *Am J Respir Crit Care Med* 2005; **172**: 128-35.

60. Sreenivasa Rao B, Seshasayana A, Pardha Saradhi SV et al. Correlation of "in vitro" release and "in vivo" absorption characteristics of rifampicin from ethylcellulose coated nonpareil beads. *Int J Pharm* 2001; **230**: 1-9.

61. Sturkenboom MG, Mulder LW, de Jager A et al. Pharmacokinetic Modeling and Optimal Sampling Strategies for Therapeutic Drug Monitoring of Rifampin in Patients with Tuberculosis. *Antimicrob Agents Chemother* 2015; **59**: 4907-13.

62. Sturkenboom MG, Akkerman OW, van Altena R et al. Dosage of isoniazid and rifampicin poorly predicts drug exposure in tuberculosis patients. *Eur Respir J*. England, 2016; 1237-9.

63. te Brake LH, Ruslami R, Later-Nijland H et al. Exposure to total and protein-unbound rifampin is not affected by malnutrition in Indonesian tuberculosis patients. *Antimicrob Agents Chemother* 2015; **59**: 3233-9.

64. Tostmann A, Mtabho CM, Semvua HH et al. Pharmacokinetics of first-line tuberculosis drugs in Tanzanian patients. *Antimicrob Agents Chemother* 2013; **57**: 3208-13.

65. van Crevel R, Nelwan RH, Borst F et al. Bioavailability of rifampicin in Indonesian subjects: a comparison of different local drug manufacturers. *Int J Tuberc Lung Dis* 2004; **8**: 500-3.

66. van Oosterhout JJ, Dzinjalamala FK, Dimba A et al. Pharmacokinetics of Antituberculosis Drugs in HIV-Positive and HIV-Negative Adults in Malawi. *Antimicrob Agents Chemother* 2015; **59**: 6175-80.

67. Weiner M, Peloquin C, Burman W et al. Effects of tuberculosis, race, and human gene SLCO1B1 polymorphisms on rifampin concentrations. *Antimicrob Agents Chemother* 2010; **54**: 4192-200.

68. Yunivita V, Dian S, Ganiem AR et al. Pharmacokinetics and safety/tolerability of higher oral and intravenous doses of rifampicin in adult tuberculous meningitis patients. *Int J Antimicrob Agents* 2016; **48**: 415-21.

69. Zhu H, Guo SC, Hao LH et al. Relative bioavailability of rifampicin in four Chinese fixed-dose combinations compared with rifampicin in free combinations. *Chin Med J (Engl)* 2015; **128**: 433-7.

70. Zwolska Z, Augustynowicz-Kopeć E, Niemirowska-Mikulska H. The pharmacokinetic factors and bioavailability of rifampicin, isoniazid and pyrazinamid fixed in one dose capsule. *Acta Pol Pharm* 2002; **59**: 448-52.
